# Association of multidimensional biomarkers of immune, nutritional, inflammatory, and metabolic status with postoperative outcomes in older hip fracture patients: a systematic review and meta-analysis

**DOI:** 10.3389/fnut.2026.1881336

**Published:** 2026-08-19

**Authors:** Yuejiao Sun, Lei Wang, Lingling Sun, Yuetao Tang, Min Lei, Chunxia Zhou, Chunhua Guo

**Affiliations:** 1 School of Nursing, Hebei Medical University, Shijiazhuang, China; 2 Department of Orthopedic Surgery, Hebei Medical University Third Hospital, Shijiazhuang, China; 3 Department of Cardiology, Hebei Medical University Third Hospital, Shijiazhuang, China; 4 Center for Integrated Traditional Chinese and Western Medicine for Preventive Treatment, Hebei Medical University Third Hospital, Shijiazhuang, China

**Keywords:** aged, biomarkers, hip fracture, inflammation, nutritional status, prognosis

## Abstract

**Background:**

The high mortality and disability rates following hip fractures in the elderly have attracted widespread attention. Underlying inflammatory, immunological, nutritional, and metabolic imbalances are major contributing factors. However, the prognostic value of multidimensional composite biomarkers encompassing these dimensions remains unclear. This systematic review aimed to evaluate the prognostic value of these composite biomarkers for postoperative outcomes in elderly patients with hip fractures.

**Methods:**

A systematic search was conducted in PubMed, Web of Science, Embase, Scopus, the Cochrane Library, ProQuest, and ClinicalTrials.gov, with a cutoff date of 7 January 2026, to identify eligible cohort studies. Data were analyzed using random-effects models for both continuous and cutoff-based categorical variables. Sensitivity analyses were conducted using the modified Knapp-Hartung method with an *ad hoc* variance correction and a Bayesian hierarchical model. The quality of the evidence was assessed using the GRADE criteria.

**Results:**

A meta-analysis was conducted, incorporating 46 cohort studies involving a total of 28,605 patients. The results showed that baseline immune-inflammatory, nutritional, and metabolic imbalances are associated with the risk of adverse postoperative outcomes. For short-term outcomes, among binary biomarkers, a low prognostic nutritional index (OR = 3.29, 95% CI: 2.22–4.88), a high neutrophil-to-lymphocyte ratio (OR = 5.17, 95% CI: 2.99–8.92), and a high glucose-to-albumin ratio (OR = 2.31, 95% CI: 1.86–2.88) constituted the core risk prognostic indicators. In the analysis of continuous variables, the prognostic nutritional index (OR = 1.09, 95% CI: 1.04–1.15), the C-reactive protein to albumin ratio (OR = 1.33, 95% CI: 1.19–1.47), and the neutrophil-to-lymphocyte ratio (OR = 1.08, 95% CI: 1.03–1.13) were also associated with an increased short-term risk. Among binary biomarkers evaluating the prognosis of medium-to-long-term adverse outcomes, in addition to the C-reactive protein to albumin ratio (OR = 3.19, 95% CI: 2.20–4.62) and the systemic immune-inflammation index (HR = 2.00, 95% CI: 1.37–2.91), the geriatric nutritional risk index (OR = 2.95, 95% CI: 1.94–4.48) also demonstrated medium-to-long-term prognostic value.

**Conclusion:**

Overall, composite biomarkers of immunity, inflammation, nutrition, and metabolism demonstrate prognostic associations with short-term and medium-to-long-term adverse outcomes in older patients with hip fractures, with core prognostic indicators predominantly supported by moderate- to high-certainty evidence. These findings support the use of these multidimensional indicators as accessible and reliable assessment tools to assist clinicians in personalized perioperative risk screening and precision management.

## Graphical abstract

**Figure:**
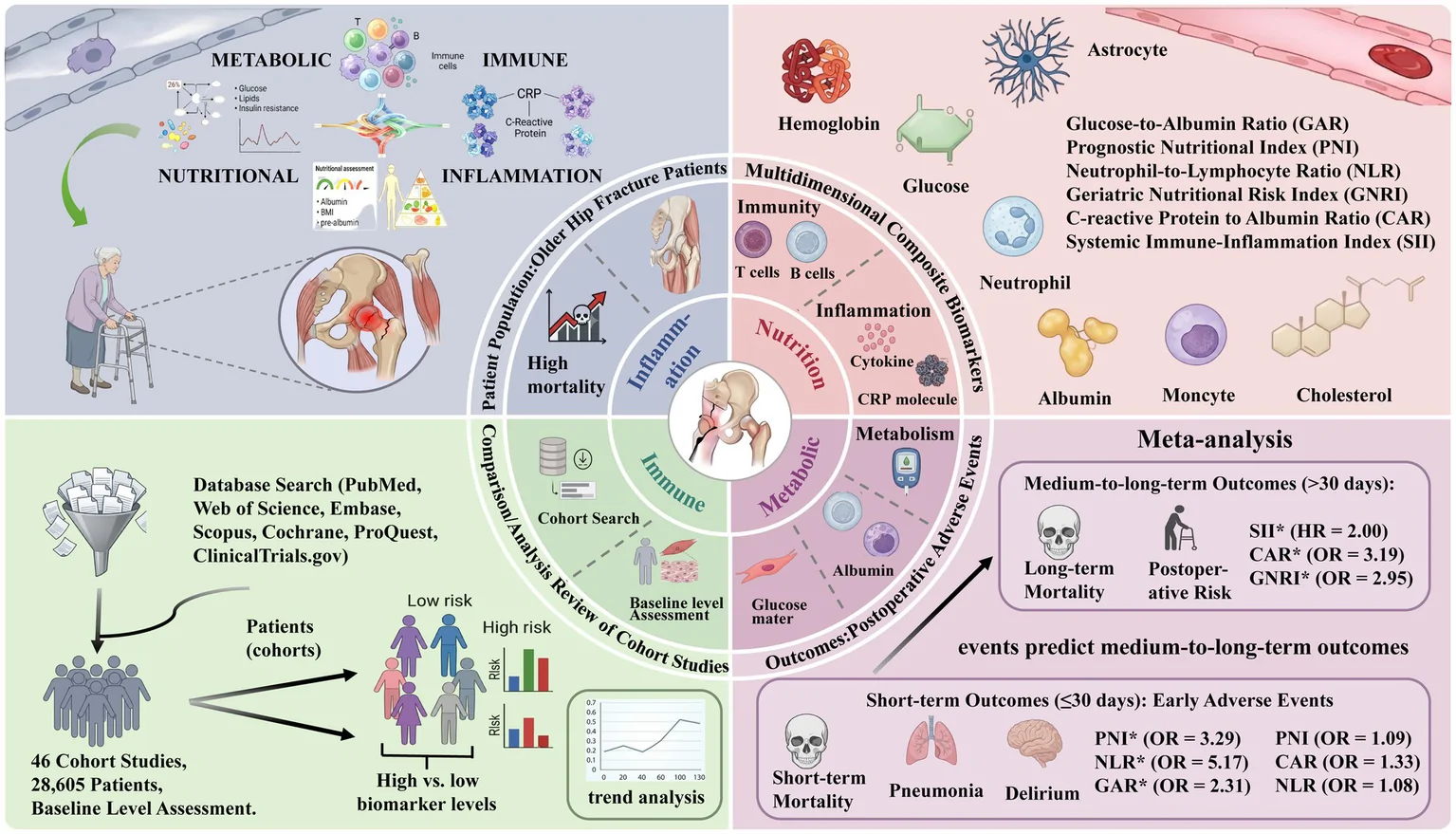

## 1 Introduction

Hip fractures in older adults represent a severe global public health burden (1, 2). Driven by rapid global aging, the annual incidence of these fractures is projected to reach 6.26 million by 2050 (3, 4). Furthermore, older patients undergoing hip fracture surgery are highly vulnerable to adverse postoperative events. Specifically, in-hospital mortality ranges from 2 to 15% (5, 6), 30-day complication rates vary between 0.98 and 7.01% (7), and 1-year mortality reaches 10–30% (8–10). Additionally, these patients often present with multiple comorbidities and varying degrees of frailty. These baseline vulnerabilities, when compounded by fracture-induced immune-inflammatory imbalance and rapid nutritional depletion, collectively drive adverse outcomes (11–13).

Composite blood biomarkers are indices derived from two or more standard peripheral blood parameters. Compared to single markers, they more comprehensively reflect the interaction between inflammatory responses and nutritional depletion under acute traumatic stress, thereby assisting in clinical risk assessments (14). Due to their accessibility, low cost, and stable prognostic performance, these markers are widely used in clinical practice (15, 16). Mechanistically, they are categorized into three types: immune and systemic inflammatory indices, such as the neutrophil-to-lymphocyte ratio (NLR); inflammatory-nutritional indices, such as the prognostic nutritional index (PNI); and basic nutritional and metabolic indices, such as the glucose to albumin ratio (GAR) and the geriatric nutritional risk index (GNRI). Clarifying their multidimensional prognostic value is crucial for optimizing early risk stratification and personalized interventions in high-risk patients (17).

However, existing systematic reviews and meta-analyses exhibit several methodological limitations. Previous studies (18) have largely been limited to single-dimensional biomarkers, failing to systematically reflect the complex interactions among inflammation, immunity, nutrition, and metabolism. Moreover, they frequently use all-cause mortality as the sole endpoint, overlooking severe perioperative complications. Second, certain studies (19) inappropriately pool continuous and categorical biomarker variables, leading to bias in baseline risk assessment. Additionally, due to the limited number of includable studies for specific biomarkers, some studies (20) rely solely on traditional meta-analysis models, which are prone to yielding false-positive results when analyzing small sample sizes. To our knowledge, no prior studies have systematically evaluated the prognostic value of multidimensional composite biomarkers for short-term and medium-to-long-term adverse outcomes in older adults with hip fractures, nor have they assessed evidence certainty using the GRADE framework.

To improve prognostic guidance, this systematic review and meta-analysis synthesizes current evidence regarding the impact of distinct composite biomarkers on adverse surgical outcomes in older patients with hip fractures. By applying the GRADE framework, our primary objective is to evaluate the prognostic associations of these biomarkers. Ultimately, we aim to establish a reliable evidence base that informs early clinical risk stratification and supports future personalized interventions for frail older adults.

## 2 Materials and methods

This study followed the Preferred Reporting Items for Systematic Reviews and Meta-Analyses (PRISMA) 2020 statement (21). The study protocol was registered in the International Prospective Register of Systematic Reviews (accessed on 14 January 2026, registration number: CRD420261285266).^1^

### 2.1 Search strategy

Seven databases were systematically queried: PubMed, Web of Science, Embase, Scopus, the Cochrane Library, ProQuest, and ClinicalTrials.gov. The core search strategy utilized Boolean operators to combine primary concepts, structured broadly as: (“Hip Fractures” OR “Femoral Neck Fractures” OR “trochanteric fracture” OR “intertrochanteric fracture” OR “subtrochanteric fracture”) AND (“Aged” OR “Older Adults” OR “Geriatric”) AND (“Biomarkers” OR “Biomarker”). Databases were searched from inception to 7 January 2026. Detailed search strategies are provided in Supplementary material. Additionally, we manually screened the reference lists of included studies and relevant reviews.

### 2.2 Eligibility criteria

Inclusion criteria were as follows: (1) evaluated the prognostic value of serum composite biomarkers for adverse postoperative outcomes in older adults with hip fractures; (2) prospective or retrospective cohort design; (3) included patients aged ≥60 years undergoing hip fracture surgery; and (4) published in English.

Exclusion criteria were: (1) multiple, pathological, periprosthetic, or non-hip fractures; (2) failure to provide sufficient data to directly extract or indirectly calculate the odds ratio (OR), hazard ratio (HR), or relative risk (RR) with their 95% confidence intervals (CI); (3) categorization as non-original research (e.g., reviews, meta-analyses, animal studies, conference abstracts, commentaries, letters, case reports, duplicate publications, or articles for which the full text was unavailable).

### 2.3 Outcomes

Serum composite biomarkers were included if they were reported in at least two independent studies, while quantitative analysis required at least three studies reporting consistent variables and effect sizes. Given the diverse clinical endpoints, outcomes were *a priori* categorized into two composite endpoints: short-term (≤30 days) and medium-to-long-term (>30 days). Short-term outcomes included early adverse events, such as short-term mortality and complications (e.g., pneumonia and delirium). Medium-to-long-term outcomes encompassed mid-term and long-term mortality and postoperative functional ability. This categorization facilitated a comprehensive evaluation of overall prognosis. Detailed outcome metrics for each biomarker are provided in Supplementary Table S1.

### 2.4 Data extraction and processing

Following the removal of duplicate publications using EndNote, literature screening, data extraction, and study quality appraisal were performed strictly in duplicate by two independent researchers. Discrepancies during these processes were resolved through mutual consensus or adjudication by a third independent investigator. In cases of overlapping datasets or patient cohorts from the same institution, only the study with the largest sample size or the most comprehensive outcome data was retained for quantitative synthesis. Extracted data included: first author’s last name, year of publication, country, study design, sample size, patient age, proportion of females, fracture type, biomarkers, outcomes, effect sizes and 95% confidence intervals, and cut-off values.

When studies reported multiple effect sizes, those adjusted for the maximum number of confounders were selected. To ensure statistical independence, each study contributed only one effect size to the meta-analysis. For multiple short-term outcomes, a predefined selection priority was applied: mortality over complications, and preoperative over postoperative metrics. For protective factors (e.g., PNI), the effect estimates and CIs were reciprocally transformed to ensure consistent effect directions. If studies lacked effect sizes or required conversion to ORs, crude ORs and 95% CIs were calculated from reconstructed 2 × 2 contingency tables when sufficient data were available (22, 23). Otherwise, these data were excluded or narratively described.

### 2.5 Quality assessment

The Quality in Prognosis Studies (QUIPS) tool (24) was utilized to gage the risk of bias. Criteria were modified from the QUIPS framework based on the methodology of Bui et al. (3) (Supplementary material). To ensure robust inter-rater reliability, the two independent researchers piloted the criteria on 25% of included studies before formal appraisal to resolve ambiguities and calibrate the assessment standards. Subsequently, an appraisal of the risk of bias was conducted for each eligible article utilizing the customized evaluation framework. Furthermore, the certainty of evidence for the prognostic value of biomarkers was downgraded by one level in the GRADE “Risk of Bias” domain if high-risk studies contributed to >60% of the cumulative statistical weight.

Studies requiring reconstructed 2 × 2 tables for crude estimates were assigned a high risk of bias in the confounding domain due to a lack of adjustment. In the statistical analysis and reporting domain, studies were rated as moderate risk if they provided detailed statistical descriptions, used appropriate regression models, and comprehensively reported results. Otherwise, they were rated as high risk. Since this rating approach could potentially introduce statistical bias, we analyzed its impact in a sensitivity analysis.

### 2.6 Statistical analysis

Biomarker data were evaluated as continuous or dichotomized variables. Effect sizes were pooled using a random-effects model (25). Heterogeneity was quantified using Cochran’s *Q* and *I*^2^ statistics (26), with results visualized in forest plots. For biomarkers with limited studies, the modified Knapp-Hartung method with an *ad hoc* variance correction (27) was applied to yield conservative confidence intervals. Additionally, a Bayesian hierarchical model (28, 29) (Supplementary material) was utilized to verify result stability in small samples. This framework was specifically chosen because it robustly handles variance in limited datasets and provides intuitive probability intervals, making complex meta-analytic results more accessible for clinical interpretation.

A leave-one-out sensitivity analysis was conducted to assess the influence of individual studies on pooled estimates. Furthermore, to test robustness across statistical assumptions, pooled estimates from random- and fixed-effects models were compared. Subgroup analyses were performed to explore potential sources of heterogeneity.

Publication bias was addressed using the non-parametric trim-and-fill method (30) for each biomarker. Following Cochrane recommendations (31), funnel plots, Begg’s (32), and Egger’s (33) tests were applied for biomarkers with ≥10 studies.

All statistical analyses were performed using R version 4.5.2 via the following packages: metafor (34), brms (35), ggplot2 (36), and robvis (37).

### 2.7 Certainty of evidence assessment

Evidence certainty for each biomarker was assessed using the adapted GRADE framework for prognostic studies (38). Under this framework, longitudinal observational studies were initially rated as high-quality evidence. Certainty was downgraded based on six domains (phase of investigation, study limitations, inconsistency, indirectness, imprecision, and publication bias) and upgraded for two domains (moderate/large effect size and dose effect). Final evidence certainty was graded as high, moderate, low, or very low (Supplementary material).

## 3 Results

### 3.1 Literature search and study selection

**Figure 1:**
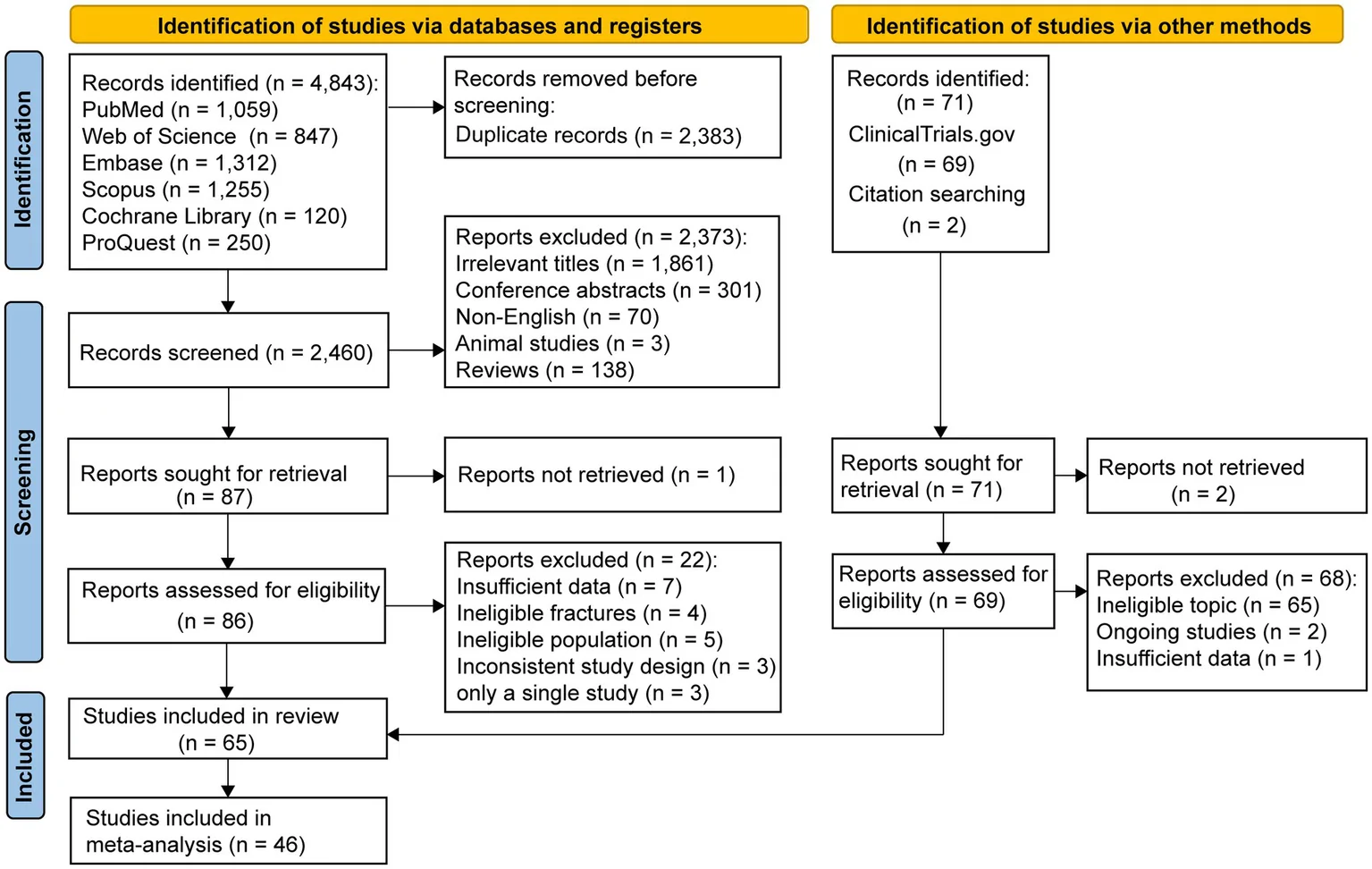
Flow diagram illustrating the systematic literature search regarding the correlation between composite biomarkers and clinical outcomes among older patients sustaining hip fractures.

The initial search yielded 4,843 records from databases and 71 from other sources. After removing 2,383 duplicates, 2,460 database records underwent title and abstract screening, resulting in 2,373 exclusions. Full texts were sought for the remaining 87 database records (one unretrieved) and 71 other records (two unretrieved). After full-text review, 22 databases and 68 other records were excluded. Ultimately, 65 studies met the inclusion criteria for the systematic review, with 46 providing sufficient data for meta-analysis. The screening workflow is detailed in Figure 1.

Detailed characteristics of the 65 included studies are provided in Table 1. Most studies were published within the past 5 years. Only one study (39) was published in 2017, and another (40) was published in 2019. Most originated from China and Turkey. Fifty-three studies used a retrospective cohort design, and 12 were prospective. All populations comprised older adults aged ≥60 years. Specifically, three studies (39, 41, 42) enrolled patients aged ≥70 years, and one (43) enrolled patients aged ≥78 years. Furthermore, most participants were female.

**Table 1:** Characteristics of studies included in the systematic review and meta-analysis.

Author (year) country[Ref]	Study design	Sample	Age (mean age)	Sex (M%)	Fracture type	Sampling time biomarker	Outcome	Effect (95%CI)	Unadjusted effect (95%CI)	Calculated from contingency table	Cut off	Risk of bias
Aydin (2023) Turkey (67)	Retrospective	343	≥65(80.81 ± 7.57)	61.8%	Hip fracture	Pre- NLR	ICU admission	OR 1.03(0.99–1.08)^a^			NA	L
						CAR	ICU admission 1-year mortality	1.37(1.09–1.72)^a^ 3.92(2.62–5.88)^a^				
Bala (2022) Turkey (92)	Retrospective	176	>65(81.36 ± 9.22)	67.6%	Hip fracture	Pre- NLR SII PLR	1-year mortality	HR 1.03(0.90–1.17) 1.00(0.92–1.08) 1.00(0.99–1.01)			NA	L
						Post- NLR 2nd SII 2nd PLR 2nd		1.07(0.99–1.17) 0.99(0.96–1.03) 1.00(0.99–1.01)				
						Post- NLR 5th SII 5th PLR 5th		1.04(1.01–1.07) 1.02(1.00–1.05) 1.00(1.00–1.01)				
Kadir (2024) Turkey (61)	Retrospective	222	>65(80.60 ± 7.40)	66.2%	Hip fracture	Pre- PNI	ICU admission in-hospital mortality	OR 1.36(1.17–1.57) 1.16(0.93–1.44)			NA	L
Cacciola (2023) Italy (71)	Retrospective	676	≥65(82.30 ± 10.30)	55.9%	Hip fracture	Pre- CAR	30-day mortality		OR 9.38(4.50–19.50)		14.72	H
Cao (2025) China (77)	Retrospective	202	≥65 (Median81)	69.0%	Hip fracture	Pre- HRR	POD	OR 0.50(0.34–0.75)			NA	L
Sercan (2020) Turkey (68)	Retrospective	254	≥65(78.74 ± 6.88)	58.7%	Hip fracture	Adm- CAR	1-year mortality	OR 3.52(1.49–8.30)			0.25	M
Zekeriya (2024) Turkey (105)	Retrospective	311	≥65(80.70 ± 8.00)	67.8%	Hip fracture	Post- SII 5th	1-year mortality	HR 2.16(1.38–3.38)			1751.9	L
Aytek (2023) Turkey (94)	Retrospective	522	≥65(78.43 ± 9.08)	66.1%	Femoral neck fracture	Post- NLR 3rd	30-day mortality	OR 1.05(1.01–1.09)			NA	M
Chen (2024) China (60)	Prospective	705	≥65(78.20 ± 7.50)	70.8%	Hip fracture	Adm- PNI	Unassisted ambulation: 120-day mobility	OR 1.69(1.10–2.61)			46.8	H
Chen (2024) China (59)	Prospective	942	≥65(79.60 ± 7.80)	72.3%	Hip fracture	Adm- PNI	3-year mortality	HR 0.39(0.28–0.55)			45.275	L
Chen (2024) China (82)	Prospective	760	≥65(79.40 ± 7.80)	71.1%	Hip fracture	Adm- MLR	3-year mortality	NA		OR2.09(1.46–3.00)	0.581 NA	L
						Adm- NLR PLR SII		HR 1.00(0.97–1.04) 1.00(1.00–1.00) 1.00(1.00–1.00)				
Choi (2025) China (75)	Retrospective	775	≥65(84.08 ± 7.88)	70.2%	Hip fracture	Pre- RAR	3-month mortality 6-month mortality 1-year mortality	HR 3.37(1.75–6.50) 3.47(2.14–5.62) 3.94(2.62–5.90)			0.468	H
Hiroto (2022) Japan (109)	Retrospective	1,040	≥65(84.70 ± 7.80)	76.7%	Hip fracture	Adm- GNRI	30-day mortality	OR 27.10(8.57–85.90)			75.4	L
Kai (2025) China (91)	Prospective	565	≥65(78.90 ± 7.80)	73.1%	Hip fracture	Adm- NLR	1-year mortality 1-year-walking independence	OR 1.13(1.09–1.17) 1.37(1.26–1.50)			NA	L
						SII	1-year-walking independence	1.00(1.00–1.00)				
Mohammad (2023) Australia (99)	Retrospective	834	≥65(84.10 ± 7.55)	71.6%	Hip fracture	Adm- NLR	in-hospital mortality	OR 1.04(0.98–1.10)^a^			NA	L
He (2020) China (83)	Prospective	780	≥65(73.33 ± 7.66)	51.4%	Hip fracture	Adm- NLR	POD	OR 3.93(2.47–6.25)			3.5	L
Hong (2023) Korea (63)	Retrospective	199	>65 (Mean80.85)	65.3%	Hip fracture	Pre- NLR PNI	in-hospital complications	OR(NLR) 1.14(1.06–1.23)	OR(PNI) 1.06(1.01–1.10)^a^		NA	L
Selami (2022) Turkey (96)	Retrospective	190	82.80 ± 6.10	66.8%	Femoral neck fracture	Post- NLR 5th	1-year mortality	OR 17.77(3.55–144.13)			5	M
Zhao (2024) China (88)	Retrospective	1,242	≥60 (Median81)	66.7%	Hip fracture	Adm- NLR	POD	OR 7.79(5.35–11.34)			7.613	L
Yuan (2024) China (78)	Retrospective	307	≥65(81.21 ± 7.61)	66.5%	Intertrochan-teric fracture	Post- HRR	AKI	OR 0.59(0.37–0.93)			NA	M
Yao (2023) China (89)	Retrospective	1,199	≥60(74.77 ± 9.61)	60.1%	Hip fracture	Adm- NLR PLR SII	POP	OR 1.20(1.01–1.42) 1.84(1.17–2.88) 1.19(1.00–1.43)			NA	L
Xu (2025) China (56)	Retrospective	260	≥65	59.6%	Femoral neck fracture	Pre- PNI	POD	OR 6.28(3.12–12.66)			45.45	L
Xing (2020) China (57)	Prospective	163	≥65 (Median72)	57.1%	Hip fracture	Pre- PNI	POD	OR 2.88(1.25–6.64)			47.45	L
Wu (2025) China (55)	Retrospective	577	≥60 (Median82)	67.9%	Hip fracture	Adm- GNRI PNI	1-year mortality	HR(PNI) 1.65(0.91–2.99)		OR(GNRI) 2.65(1.51–4.66)	98 38	H
Wu (2025) China (54)	Retrospective	526	≥60 (Median73)	70.0%	Hip fracture	Pre- PNI	BADL dependence at 2 years	OR 0.90(0.82–0.99)			NA	H
Wang (2021) China (107)	Retrospective	460	≥60 (Mean79.31)	67.0%	Hip fracture	Adm- PLR	1-year mortality	HR 1.55(1.01–2.39)			189	L
Wang (2025) China (73)	Retrospective	1,707	≥60 (Median80)	64.5%	Hip fracture	Adm- HALP	90-day mortality	HR 0.99(0.98–1.00)			NA	L
Wang (2021) China (104)	Prospective	290	≥60(77.60 ± 8.60)	65.2%	Hip fracture	Adm- SII	1-year mortality	HR 1.08(1.01–1.17)			NA	L
Wang (2025) China (87)	Retrospective	1,807	≥60 (Median82)	59.9%	Hip fracture	Pre- GAR NLR	POD	OR 2.40(1.60–3.70) 1.00(1.00–1.10)			0.2 NA	L
Wang (2024) China (112)	Retrospective	1,231	≥60(74.99 ± 9.47)	60.2%	Hip fracture	Adm- GAR	UTIs	OR 2.44(1.69–3.54)			0.18	L
Vural (2025) Turkey (70)	Retrospective	435	≥65(81.56 ± 7.37)	68.3%	Hip fracture	Adm- HALP CAR	1-year mortality		OR 11.79 (7.19–19.34) 4.14 (2.69–6.38)		17.975 0.6574	H
Turgut (2022) Turkey (51)	Retrospective	335	≥70(83.21 ± 6.10)	59.4%	Hip fracture	Per- NLR	90-day mortality	OR 1.05(1.01–1.09)			NA	M
Mahmut (2024) Turkey (52)	Prospective	124	≥70(80.40 ± 7.19)	62.1%	Femoral neck fracture	Pre- PNI	1-year mortality	HR 1.24(1.05–1.46)^a^			NA	H
Tsutsui (2023) Japan (110)	Prospective	623	≥60 (Mean82)	79.9%	Hip fracture	Adm- GNRI	5-year mortality			OR 3.42(2.41–4.85)	92	H
Tang (2024) China (111)	Retrospective	1,279	≥60(74.70 ± 9.55)	60.3%	Hip fracture	Pre- GAR	30-day POP	OR 2.14(1.50–3.05)			0.175	L
Ren (2017) China (49)	Prospective	80	≥70(86.00 ± 5.00)	56.3%	Hip fracture	Pre- PNI	1-year mortality	HR 0.20(0.03–0.65)			NA	L
Perna (2025) Italy (98)	Retrospective	395	≥65(84.00 ± 12.30)	56.4%	Hip fracture	Adm- NLR PLR	1-year mortality	HR(PLR) 1.81(1.61–2.23)		OR(NLR) 2.15(1.11–4.19)	7.2189.4	H
Mi (2024) China (58)	Prospective	369	≥65 (Mean78.8)	28.5%	Hip fracture	Pre- PNI	POD	OR 1.08(1.00–1.16)^a^			NA	L
Omer (2023) Israel (100)	Retrospective	1,551	≥65(90.76 ± 1.88)	68.7%	Hip fracture	Adm- NLR	1-year mortality	HR 1.02(1.00–1.05)			NA	L
Long (2023) China (66)	Retrospective	1,085	≥60 (Median78)	66.4%	Hip fracture	Adm- NLR MLR CAR	1-year mortality			OR 1.65(1.13–2.41) 2.37(1.61–3.48) 2.45(1.68–3.58)	7.28 0.76 1.36	H
Liu (2025) China (85)	Retrospective	1,238	≥60(74.83 ± 9.59)	60.5%	Hip fracture	Adm- NLR PLR SII	UTIs	OR 1.30(1.12–1.52) 1.20(1.04–1.38) 1.31(1.13–1.52)			NA	L
Li (2025) China (84)	Retrospective	193	82.90 ± 9.05	61.1%	Intertrochan-teric fracture	Post- SII NLR	POP	OR 1.00(1.00–1.00) 1.28(1.08–1.52)			NA	L
Kolhe (2025) United Kingdom (101)	Retrospective	1,039	82.50 ± 8.10	69.2%	Hip fracture	Adm- NLR MLR	30-day mortality	OR 1.37(0.96–1.95) 1.46(0.96–2.23)			NA	L
Kim (2023) South Korea (72)	Retrospective	300	≥65(79.90 ± 7.60)	72.7%	Hip fracture	Pre- CAR	POD		OR 1.31(1.15–1.49)		NA	H
Fujimoto (2022) Japan (53)	Retrospective	108	≥78 (Median84)	78.7%	Hip fracture	Pre- GNRI	1-year mortality	OR 1.25(1.08–1.47)^a^			NA	L
Ji (2023) China (90)	Retrospective	315	≥60(74.31 ± 6.75)	46.7%	Intertrochan-teric fracture	Pre- NLR	POD	OR 3.49(1.73–5.98)			4.85	M
Balta (2022) Turkey (69)	Retrospective	165	≥60(83.09 ± 8.52)	57.6%	Intertrochan-teric fracture	Pre- CLR CAR	30-day mortality	OR 1.01(0.99–1.02) 1.33(0.99–1.79)			NA	M
Cheng (2023) China (79)	Prospective	1,958	≥60 (Median76)	67.0%	Hip fracture	Pre- CONUT	Loss of mobility (180 days)	RR 1.42(1.12–1.80)			5	L
Fatih (2025) Turkey (74)	Retrospective	60	≥65(83.20 ± 6.30)	46.7%	Femoral neck fracture	Adm- HALP	6-month mortality	OR 1.09(1.06–1.13)			NA	L
Chen (2024) China (80)	Retrospective	303	≥65 (Median82)	69.0%	Hip fracture	Adm- CONUT	1-year mortality	HR 2.32(1.24–4.36)			5	L
Chua (2025) Taiwan (106)	Retrospective	159	≥60(80.70 ± 8.99)	55.3%	Hip fracture	Pre- GNRI SII CLR	1-year mortality		HR 0.95(0.91–0.98) 1.00(1.00–1.00) 1.01(0.98–1.03)		NA	H
Coşkun (2025) Turkey (76)	Retrospective	532	≥65 (Median81)	67.5%	Hip fracture	Pre- RAR	in-hospital mortality 3-month mortality 1-year mortality	OR 1.45(1.13–1.87) 1.66(1.34–2.06) 2.09(1.66–2.63)			NA	L
Zhou (2024) China (102)	Retrospective	597	≥60(77.55 ± 10.90)	78.4%	Hip fracture	Adm- SII GNRI	1-year mortality		HR(SII) 2.69(1.78–4.07)	OR(GNRI) 4.88(2.90–8.21)	1621.92 92	H
Bingöl (2020) Turkey (93)	Retrospective	241	≥65(81.09 ± 8.11)	60.6%	Hip fracture	Adm- NLR MLR	1-year mortality			OR 8.26(4.19–16.28) 13.09(6.61–25.92)	6.55 0.635	H
Kekeç (2022) Turkey (95)	Retrospective	317	≥65 (Mean81.2)	53.3%	Intertrochan-teric fracture	Pre- NLR	ICU admission			OR 2.70(1.70–4.28)	6.14	H
Faust (2023) Germany (64)	Retrospective	156	≥65 (Mean83.4)	77.1%	Intertrochan-teric fracture	Post- PNI	Limited mobility at discharge	OR 1.18(1.08–1.30)			NA	L
Temiz (2019) Turkey (50)	Retrospective	50	≥65 (Mean73.81)	100.0%	Intertrochan-teric fracture	Adm- NLR	1-year mortality			OR 23.22 (5.10–105.60)	4.7	H
Yotsuya (2024) Japan (62)	Retrospective	246	≥65 (83.00 ± 9.60)	74.0%	Hip fracture	Pre- PNI	Limited mobility at discharge		OR 3.08(1.82–5.21)		45	M
Miniksar (2021) Turkey (97)	Retrospective	188	≥65 (Median78)	59.0%	Hip fracture	Adm- NLR	ICU admission			OR 17.53(7.00–43.89)	9.65	H
Popp (2024) Austria (65)	Retrospective	1,500	>60 (Mean81.1)	70.8%	Hip fracture	Pre- PNI	1-month mortality			OR 2.41(1.43–4.07)	40	H
Zhou (2021) China (86)	Retrospective	124	≥60 (Mean76)	58.9%	Hip fracture	Post- NLR	1-year mortality			OR 18.00(6.18–52.41)	6.939	H
Yerli (2024) Turkey (108)	Retrospective	1,345	≥65(80.27 ± 7.45)	62.9%	Hip fracture	Adm- GNRI	90-day mortality			OR 1.80(1.22–2.64)	98	H
Tekin (2022) Turkey (108)	Retrospective	124	≥60 (Mean79.7)	52.4%	Intertrochan-teric fracture	Adm- MLR	1-year mortality			OR 13.13(4.23–40.72)	0.54	H
Chen (2024) China (81)	Retrospective	437	≥60 (Mean80.00)	72.2%	Hip fracture	Pre- NLR	POD	OR 1.02(0.92–1.12)			NA	L
Tan (2025) China (103)	Retrospective	2,766	≥65(79.50 ± 7.30)	69.8%	Hip fracture	Post- SII	2-year mortality	HR 1.47(1.07–2.01)			1293.03	L

[a] Indicates reversal of the effect direction. “Unadjusted effect” denotes effect sizes extracted from univariate analyses. “Calculated from contingency table” indicates effect estimates derived by reconstructing 2 × 2 contingency tables. Adm-, at admission; AKI, postoperative acute kidney injury; POD, postoperative delirium; POP, postoperative pneumonia; Post-, postoperative; Pre-, preoperative; UTIs, urinary tract infections. CI, confidence interval; OR, odds ratio; HR, hazard ratio; CAR, C-reactive protein-to-albumin ratio; GAR, glucose-to-albumin ratio; GNRI, geriatric nutritional risk index; MLR, monocyte-to-lymphocyte ratio; NLR, neutrophil-to-lymphocyte ratio; PNI, prognostic nutritional index; SII, systemic immune-inflammation index; CLR, C-reactive protein to lymphocyte ratio; PLR, platelet to lymphocyte ratio; CONUT, controlling nutritional status score; HRR, hemoglobin to red cell distribution width ratio; RAR, red cell distribution width to albumin ratio; HALP, hemoglobin, albumin, lymphocyte and platelet score. L, Low risk; M, Moderate risk; H, High risk.

**Figure 2:**
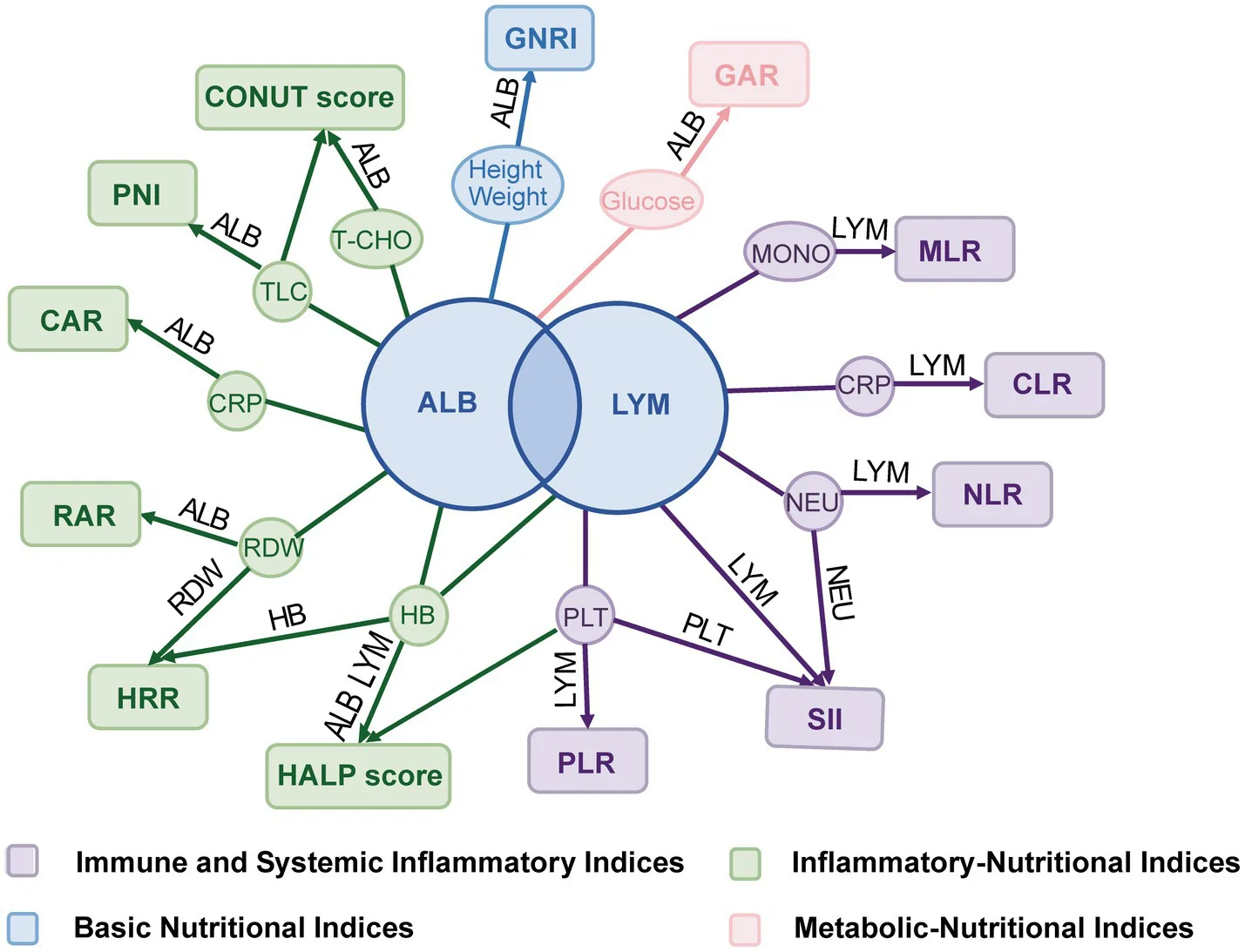
Classification and structural interrelationships of the 13 composite biomarkers. The central overlapping circles highlight albumin (ALB) and lymphocytes (LYM) as the most frequently shared core parameters among the indices. The surrounding biomarkers are color-coded according to their underlying physiological mechanisms: Immune and systemic inflammatory indices (purple), inflammatory-nutritional indices (green), basic nutritional indices (blue), and metabolic-nutritional indices (pink). Arrows indicate the specific constituent parameters for each composite biomarker. CRP, C-Reactive Protein; HB, Hemoglobin; MONO, Monocyte; NEU, Neutrophil; PLT, Platelet; RDW, Red Blood Cell Distribution Width; TCL, Total Cholesterol; TLC, Total Lymphocyte Count.

A total of 13 composite biomarkers were identified and classified into three mechanistic categories: (1) inflammatory-nutritional indices, including the prognostic nutritional index (PNI, calculated from serum albumin levels and total lymphocyte counts) (39, 42, 44–55), C-reactive protein to albumin ratio (CAR) (56–62), hemoglobin, albumin, lymphocyte and platelet score (HALP) (60, 63, 64), red cell distribution width to albumin ratio (RAR) (65, 66), hemoglobin to red cell distribution width ratio (HRR) (67, 68), and controlling nutritional status score (CONUT, derived from serum albumin, total cholesterol, and total lymphocyte count) (69, 70); (2) immune and systemic inflammatory indices, such as the neutrophil to lymphocyte ratio (NLR) (40, 41, 53, 56, 57, 71–91), systemic immune-inflammation index (SII) (72, 74, 75, 79, 81, 82, 92–96), platelet to lymphocyte ratio (PLR) (72, 75, 79, 82, 88, 97), monocyte to lymphocyte ratio (MLR) (56, 72, 83, 91, 98), and C-reactive protein to lymphocyte ratio (CLR) (59, 97); and (3) basic nutritional and metabolic indices, including the geriatric nutritional risk index (GNRI) (43, 45, 92, 97, 99–101) and glucose to albumin ratio (GAR) (77, 102, 103). The shared components and physiological interrelationships of these indices are visually summarized in Figure 2, while their exact calculation formulas are provided in Supplementary Table S2.

Most studies used baseline measurements collected at admission or preoperatively. Specifically, measurements were taken at admission (30 studies), preoperatively (26 studies), or postoperatively (nine studies).

For short-term outcomes, studies evaluated mortality (*n* = 10), postoperative complications (*n* = 16), and intensive care unit (ICU) admission (*n* = 4). For medium-to-long-term outcomes, 34 studies investigated mortality and four addressed postoperative functional capacity.

### 3.2 Risk of bias within the selected literature

**Figure 3:**
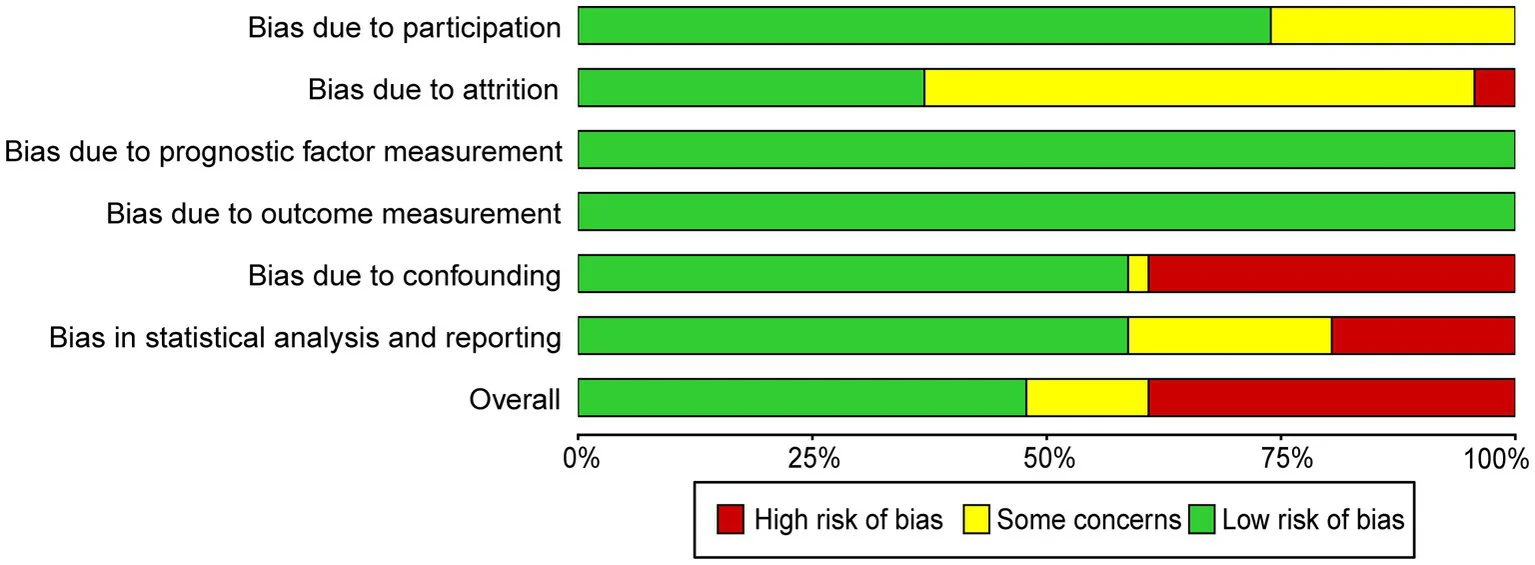
Risk of bias summary for the 46 studies incorporated into the meta-analysis.

Figure 3 shows the unweighted bias distribution across the 46 studies included in the meta-analysis. All included studies were evaluated using the QUIPS tool. Overall study quality was acceptable: 22 studies had a low risk of bias, six had a moderate risk, and 18 had a high risk. Specifically, high risk ratings were driven by loss to follow-up (*n* = 2), unadjusted confounders (*n* = 18), and statistical analysis issues (*n* = 9). Among the 14 pooled estimates, only one included a study rated as high-risk due to substantial loss to follow-up. Although the remaining studies controlled for confounders, they were conservatively classified as high-risk due to effect size conversion requirements. Detailed quality evaluations for the 65 studies in the descriptive synthesis are provided in Supplementary Figure S1.

### 3.3 Meta-analysis of various biomarkers and summary of evidence

**Figure 4:**
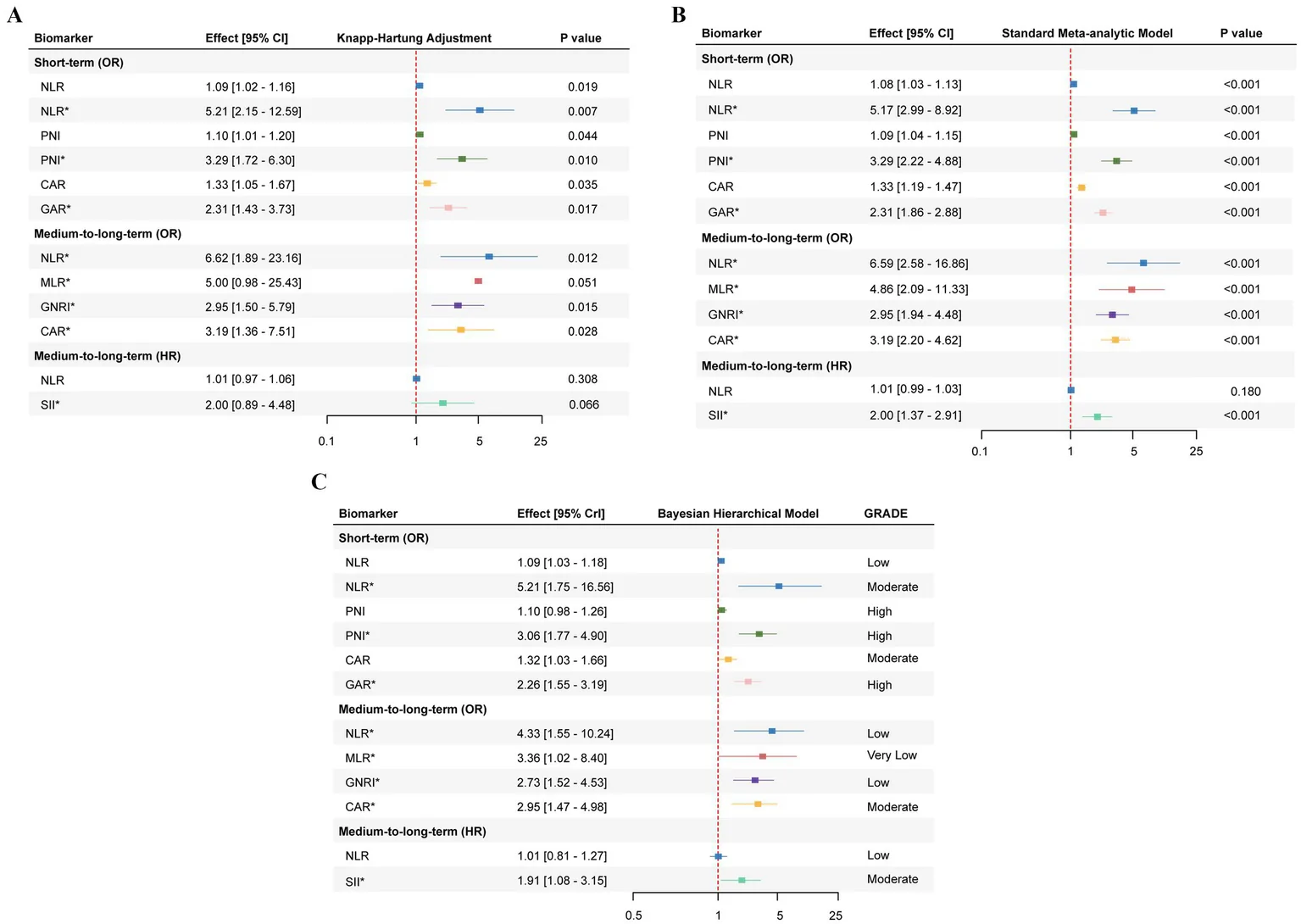
Overview of the aggregated effect estimates alongside GRADE certainty evaluations concerning composite biomarkers across different statistical models. **(A)** Summary of consolidated effect estimates and their 95% confidence intervals (CIs) derived via the conventional quantitative synthesis approach. **(B)** Adjusted pooled effect sizes, 95% CIs, and *p* values derived from the Knapp-Hartung variance adjustment method. **(C)** Pooled effect sizes coupled with 95% credible intervals (CrIs) generated through the Bayesian hierarchical framework, alongside the final GRADE certainty of evidence ratings. Those biomarkers marked with an asterisk [*] represent categorical variables classified based on their respective cutoff values, while those without an asterisk were analyzed as continuous variables. Effect sizes are presented in the form of odds ratios (ORs) or hazard ratios (HRs), along with their respective 95% confidence intervals (CIs) or 95% credible intervals (CrIs) derived from the Bayesian model. The vertically oriented red dashed line signifies the no-effect threshold (OR or HR = 1). Color coding was applied for each biomarker to facilitate visual tracking across different statistical models.

PNI, CAR, GAR, NLR, SII, MLR, and GNRI are core composite biomarkers reflecting the immune, inflammatory, nutritional, and metabolic status of older adults with hip fractures. Figure 4 illustrates the prognostic value of these biomarkers for short-term and medium-to-long-term outcomes. A random-effects model was used for the primary analysis. The modified Knapp-Hartung method and a Bayesian hierarchical model were applied for sensitivity analyses. GRADE assessments of evidence certainty are provided in Supplementary Table S3.

**Figure 5:**
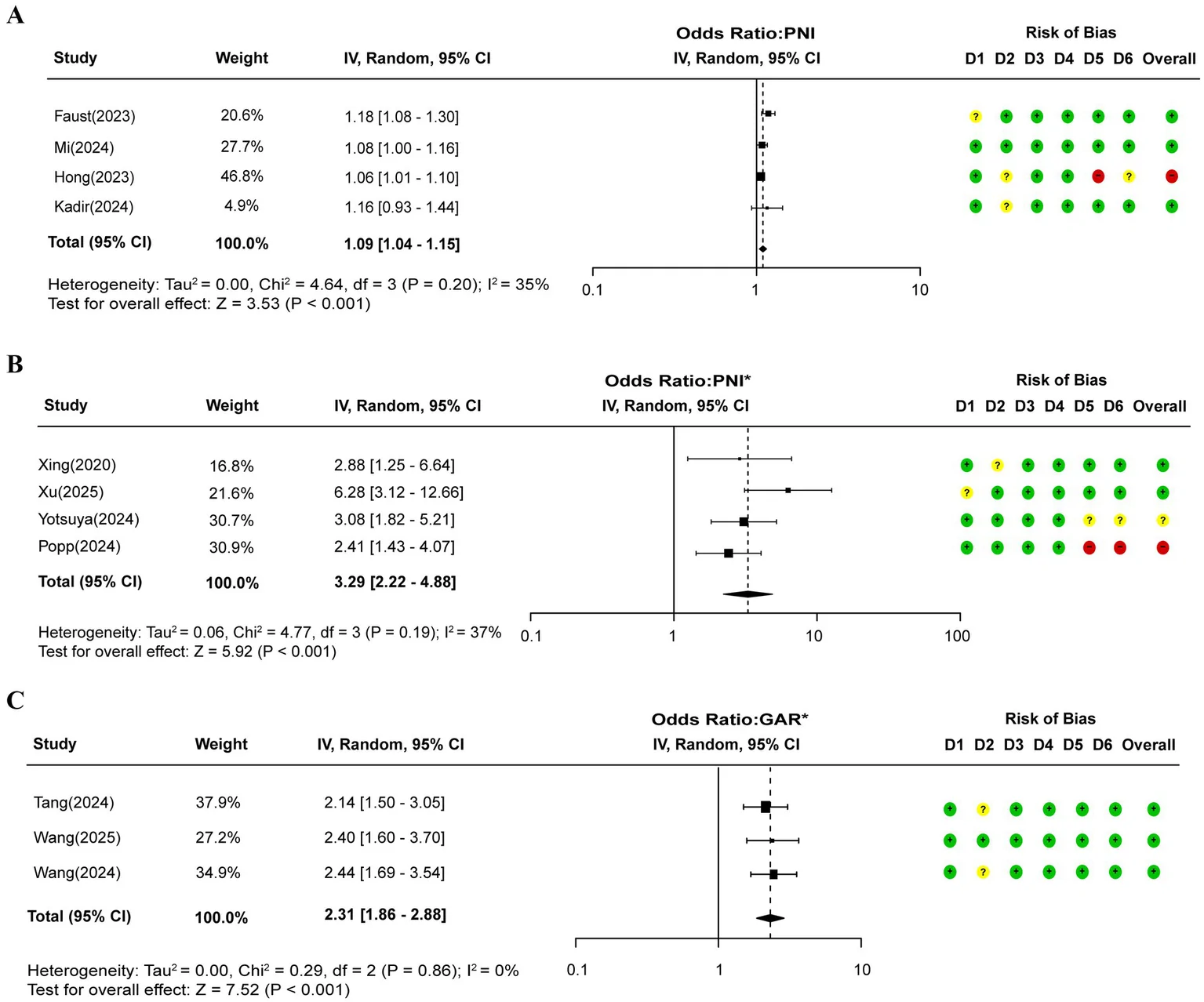
Forest plot of composite biomarkers with high-quality evidence. **(A)** Forest plot presenting the pooled odds ratio for the prognostic nutritional index (PNI). **(B)** Forest plot detailing the aggregated effect estimate for PNI*. **(C)** Forest plot displaying the combined odds ratio for the glucose to albumin ratio (GAR). The right-sided panel of each graphic demonstrates the methodological quality appraisal conducted via the Quality in Prognosis Studies (QUIPS) tool. The evaluation encompasses six specific domains: study participation (D1), attrition (D2), prognostic factor measurement (D3), outcome assessment (D4), confounding evaluation (D5), and statistical reporting (D6). Low, moderate, and elevated risks of bias are denoted by green, yellow, and red markers, respectively. Biomarkers denoted by an asterisk [*] designate categorical variables stratified according to their specific threshold values, whereas those lacking an asterisk underwent analysis as continuous variables.

Three indicators were supported by high-quality evidence. These included dichotomous PNI (OR = 3.29, 95% CI: 2.22–4.88) and continuous PNI (OR = 1.09, 95% CI: 1.04–1.15) as immunonutritional indicators, and dichotomous GAR (OR = 2.31, 95% CI: 1.86–2.88) as a metabolic-nutritional indicator. Figure 5 presents the forest plot for these high-quality biomarkers. Remaining forest plots are provided in Supplementary Figure S2.

#### 3.3.1 Neutrophil to lymphocyte ratio

NLR was reported as continuous and binary variables for both short-term and medium-to-long-term outcomes. In the assessment of short-term endpoints with NLR treated continuously, pooled analysis of 10 studies (53, 57, 71, 74, 75, 77, 79, 84, 89, 91) yielded low-quality evidence. Each unit increase elevated the risk of short-term adverse outcomes by 8% (OR = 1.08, 95% CI: 1.03–1.13). Evidence certainty was downgraded one level for inconsistency due to high heterogeneity (χ^2^ = 26.98, *p* < 0.001, *I*^2^ = 67%). After applying the trim-and-fill method, the adjusted confidence interval crossed 1.00. Consequently, certainty was further downgraded for potential reporting bias.

Regarding the evaluation of NLR as a continuous variable for medium-to-long-term outcomes, two studies (41, 81) were excluded due to incompatible and inconvertible effect sizes. Analysis of the remaining three studies (72, 82, 90) yielded low-quality evidence. Continuous NLR showed no clear association with medium-to-long-term adverse outcomes (HR = 1.01, 95% CI: 0.99–1.03, χ^2^ = 0.89, *p* = 0.64, *I*^2^ = 0%). Certainty was downgraded two levels for imprecision because both the Knapp-Hartung 95% CI (HR = 1.01, 95% CI: 0.97–1.06, *p* = 0.308) and Bayesian 95% CrI (HR = 1.01, 95% CrI: 0.81–1.27) crossed 1.00.

Alternatively, when assessing short-term outcomes using NLR as a binary variable, a meta-analysis of five studies (73, 78, 80, 85, 87) yielded moderate-quality evidence. High NLR was associated with the risk of short-term adverse outcomes (OR = 5.17, 95% CI: 2.99–8.92). Evidence certainty was downgraded one level for inconsistency due to substantial heterogeneity (χ^2^ = 21.68, *p* < 0.001, *I*^2^ = 82%).

Finally, when NLR was utilized as a binary variable for medium-to-long-term outcomes, pooled analysis of six studies (40, 56, 76, 83, 86, 88) yielded low-quality evidence. High NLR correlated with a greater risk of medium-to-long-term adverse outcomes (OR = 6.59, 95% CI: 2.58–16.86). Evidence certainty was downgraded one level for inconsistency due to high heterogeneity (χ^2^ = 40.47, *p* < 0.001, *I*^2^ = 88%). Because some effect sizes were calculated from 2 × 2 tables, studies with high risk of bias carried disproportionately large weights in the pooled estimates. This imbalance led to a further one-level downgrade in certainty.

#### 3.3.2 Systemic immune-inflammation index

SII was reported as continuous and categorical variables for both short-term and medium-to-long-term outcomes. Three investigations were incorporated into the evaluation of SII as a continuous variable to assess short-term outcomes (74, 75, 79). Because one study (74) reported an inconvertible variance (OR = 1.00, 95% CI: 1.00–1.00), a narrative synthesis was performed: two studies (74, 79) showed no association with postoperative pneumonia, whereas one (75) linked SII to urinary tract infections (OR = 1.31, 95% CI: 1.13–1.52). Thus, evidence was insufficient to link continuous SII to short-term outcomes.

For continuous SII and medium-to-long-term outcomes, four studies were identified (72, 82, 94, 96). Two studies (72, 96) lacked valid variances (OR = 1.00, 95% CI: 1.00–1.00) and were narratively described: one (94) associated SII with 1-year mortality (OR = 1.08, 95% CI: 1.01–1.17), while the others showed no clear prognostic associations. Consequently, no definitive link could be established between continuous SII and medium-to-long-term outcomes.

Conversely, when evaluating SII as a binary variable for medium-to-long-term outcomes, a meta-analysis of three studies (92, 93, 95) generated moderate-quality evidence. Elevated SII was associated with a higher risk of medium-to-long-term adverse outcomes (HR = 2.00, 95% CI: 1.37–2.91). Evidence certainty was downgraded one level for inconsistency due to substantial heterogeneity (χ^2^ = 5.57, *p* = 0.06, *I*^2^ = 64%).

#### 3.3.3 Prognostic nutritional index

PNI was reported as continuous and binary variables for short-term outcomes. A meta-analysis incorporating four studies (48, 51, 53, 54) yielded high-quality evidence upon evaluating PNI as a continuous variable. Each unit increase in PNI elevated the risk of short-term adverse outcomes by 9% (OR = 1.09, 95% CI: 1.04–1.15, χ^2^ = 4.64, *p* = 0.20, *I*^2^ = 35%).

Alternatively, a meta-analysis integrating four studies (46, 47, 52, 55) yielded high-quality evidence upon analyzing PNI as a binary variable. Low PNI was associated with an increased risk of short-term adverse outcomes (OR = 3.29, 95% CI: 2.22–4.88, χ^2^ = 4.77, *p* = 0.19, *I*^2^ = 37%).

#### 3.3.4 C-reactive protein to albumin ratio

CAR was reported as a continuous and binary variable for both short-term and medium-to-long-term outcomes. When CAR was assessed as a continuous variable for short-term outcomes, a meta-analysis of three studies (57, 59, 62) yielded moderate-quality evidence. Each unit increase in CAR elevated the risk of short-term adverse outcomes by 33% (OR = 1.33, 95% CI: 1.19–1.47, χ^2^ = 0.11, *p* = 0.95, *I*^2^ = 0%). Because effect sizes in a large study were derived from reconstructed 2 × 2 tables, studies with a high risk of bias carried over 60% of the pooled weight. This imbalance led to a one-level downgrade in certainty.

When CAR was utilized as a binary variable to assess medium-to-long-term outcomes, a meta-analysis of three studies (56, 58, 60) produced moderate-quality evidence. High CAR was associated with a higher risk of medium-to-long-term adverse outcomes (OR = 3.19, 95% CI: 2.20–4.62, χ^2^ = 3.28, *p* = 0.19, *I*^2^ = 39%). Because effect sizes from two studies were converted using 2 × 2 tables, studies with a high risk of bias again exceeded 60% of the pooled weight. Consequently, evidence certainty was downgraded one level.

#### 3.3.5 Glucose to albumin ratio

For binary GAR and short-term outcomes, meta-analysis of three studies (77, 102, 103) yielded high-quality evidence. High GAR was associated with an increased risk of short-term adverse outcomes (OR = 2.31, 95% CI: 1.86–2.88, χ^2^ = 0.29, *p* = 0.86, *I*^2^ = 0%).

#### 3.3.6 Monocyte to lymphocyte ratio

When MLR was evaluated as a binary variable to assess medium-to-long-term outcomes, a meta-analysis of four studies (48, 72, 83, 98) yielded very low-quality evidence. High MLR may be associated with an increased risk of medium-to-long-term adverse outcomes (OR = 4.86, 95% CI: 2.09–11.33). Evidence certainty was downgraded one level for inconsistency due to high heterogeneity (χ^2^ = 29.70, *p* < 0.001, *I*^2^ = 90%). Because some effect sizes were derived from 2 × 2 tables, studies with a high risk of bias carried disproportionately large weights, leading to a further one-level downgrade in certainty. After applying the trim-and-fill method, the effect size decreased by 21.38%, prompting an additional one-level downgrade for publication bias.

#### 3.3.7 Geriatric nutritional risk index

For binary GNRI and medium-to-long-term outcomes, meta-analysis of four studies (45, 92, 99, 101) yielded low-quality evidence. Low GNRI was associated with a higher risk of medium-to-long-term adverse outcomes (OR = 2.95, 95% CI: 1.94–4.48). Because some effect sizes were derived from 2 × 2 tables, studies with a high risk of bias carried disproportionately large weights, leading to a one-level downgrade in certainty. Although subgroup analyses reduced overall heterogeneity (χ^2^ = 10.69, *p* = 0.01, *I*^2^ = 72%) to 19% within the GNRI <98 and <92 strata, certainty was conservatively downgraded one level for inconsistency.

### 3.4 Sensitivity analysis and publication bias

A leave-one-out sensitivity analysis was conducted for all pooled biomarkers. The removal of individual studies did not alter the significance or direction of any pooled effect sizes. Additionally, pooled estimates from random-effects and fixed-effects models were compared. Effect directions and statistical significance remained consistent across all biomarkers regardless of the model used.

Subgroup analyses were performed for biomarkers with high heterogeneity (Supplementary Figure S3). The results showed that clinical outcome phenotypes were a significant source of heterogeneity in the continuous NLR variable for short-term outcomes (*p* < 0.001). Continuous NLR demonstrated prognostic associations with infection-related complications and mortality, but not postoperative delirium. For medium-to-long-term outcomes, unadjusted crude ORs substantially overestimated the pooled effect of MLR (*p* < 0.001). Cutoff values (92 vs. 98) also substantially affected GNRI risk estimates (*p* = 0.004).

Trim-and-fill analysis was conducted for all pooled biomarkers. For most biomarkers, effect directions remained unchanged, and effect sizes decreased by less than 20%, indicating no substantial publication bias. The effect size decreased by 21.38% only for MLR in medium-to-long-term outcomes. For continuous NLR and short-term outcomes, the adjusted 95% CI crossed 1.00, indicating potential publication bias.

**Figure 6:**
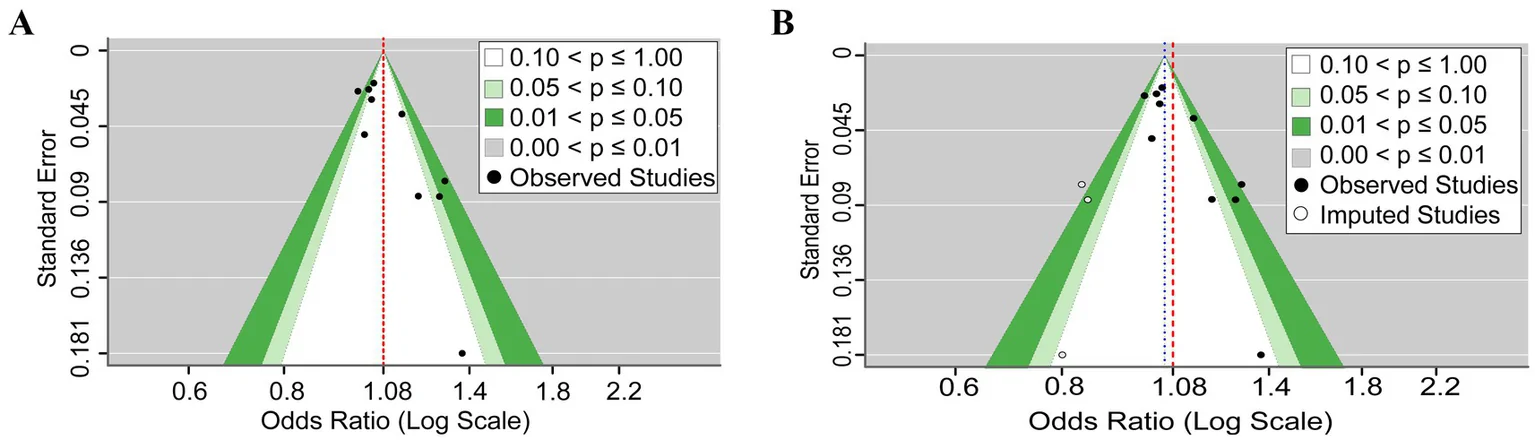
Funnel plots evaluating publication bias regarding the continuous NLR variable and short-term adverse outcomes. **(A)** Standard funnel plot of the 10 originally included studies. **(B)** Adjusted funnel plot following correction via the non-parametric trim-and-fill method. Solid black markers designate the primary investigations incorporated into the analysis. Hollow white data points signify the theoretically missing records generated via the non-parametric trim-and-fill method. The red dashed line illustrates the initial pooled effect estimate before adjustment. The blue broken line represents the adjusted pooled effect estimate.

For continuous NLR and short-term outcomes (10 studies), publication bias was assessed using funnel plots (Figure 6A), Egger’s, and Begg’s tests. Egger’s test (*p* = 0.0101), but not Begg’s test (*p* = 0.2164), indicated potential bias. Given the limited statistical power of Begg’s test, publication bias was conservatively inferred based on Egger’s test and funnel plot asymmetry. The trim-and-fill method was applied to adjust the pooled estimates. Three theoretically missing studies were imputed to restore funnel plot symmetry (Figure 6B). Consequently, the adjusted pooled effect size lost statistical significance (OR = 1.05, 95% CI: 1.00–1.10). This suggested that the initially observed statistical association was likely driven by publication bias.

### 3.5 Narrative review

Narrative review findings were generally consistent with the meta-analysis (Supplementary Table S1), reinforcing the prognostic value of NLR and PNI. For unpooled indicators, HRR showed prognostic potential for postoperative complications, while RAR, HALP, and CONUT demonstrated prognostic value for mortality, particularly medium-to-long-term mortality. Despite exclusion from quantitative synthesis due to limited studies, these parameters retain clinical relevance. Furthermore, the prognostic utility of PLR was found to be highly dependent on its data type. Continuous PLR was associated with short-term complications (e.g., POP and UTIs), whereas binary PLR correlated with medium-to-long-term prognosis (e.g., 1-year mortality). Finally, in the present review, CLR was found to have no clear association with either short-term or medium-to-long-term postoperative outcomes.

## 4 Discussion

Although previous studies have examined single-dimensional markers, this review is the first to systematically investigate multidimensional composite biomarkers based on systemic immune-inflammatory, inflammatory-nutritional, and metabolic-nutritional mechanisms in older adults with hip fractures. We evaluated the clinical prognostic value of 13 composite biomarkers (NLR, SII, MLR, PLR, CLR, PNI, CAR, RAR, HRR, HALP, CONUT, GNRI, and GAR) for postoperative adverse outcomes in this population. Following sensitivity analyses using the modified Knapp-Hartung method with *ad hoc* variance correction and a Bayesian hierarchical model, our pooled results remained largely consistent. Specifically, for short-term outcomes, both PNI (analyzed as continuous and binary variables) and GAR (analyzed as a binary variable) demonstrated prognostic utility supported by high-quality evidence, suggesting their potential as early clinical risk screening tools. For medium-to-long-term prognosis, high NLR, CAR, SII, MLR, and low GNRI suggested potential associations with poor outcomes, though these findings should be interpreted cautiously due to the moderate-to-low certainty of the evidence.

Hip fractures, as a severe traumatic stress, trigger widespread systemic inflammatory responses. Our findings indicate that elevated NLR, SII, and CAR may be associated with poor medium-to-long-term outcomes. Notably, categorical NLR based on cutoff values appeared more robust than continuous NLR in evaluating mortality risk. This robustness is supported by our finding that continuous NLR lost statistical significance after trim-and-fill adjustment for publication bias. Aligning with previous findings (18), the impact of systemic inflammation on outcomes appears non-linear (104), with adverse events likely manifesting only when inflammation exceeds physiological thresholds and overwhelms compensatory mechanisms. Pathophysiologically, these composite indices capture this breakdown: the components of NLR reflect innate immune overactivation and adaptive immune suppression (105), while increased CAR indicates an exacerbated acute-phase inflammatory burden. Together, this persistent dysregulation impedes tissue repair and increases the risk of medium-to-long-term secondary infections and organ failure (106). Conversely, although MLR showed a prognostic trend for mortality, its independent prognostic value requires further validation, as current evidence is largely limited by unadjusted confounders. Similarly, evidence regarding PLR and CLR remains limited, and they are not currently recommended as routine risk stratification tools. Clinically, rather than isolated parameters, these validated composite biomarkers offer a more comprehensive reflection of immune exhaustion, aiding physicians in identifying highly vulnerable patients early.

Albumin-based composite indices demonstrated prognostic utility for short-term adverse outcomes, with PNI supported by high-quality evidence and CAR supported by moderate-quality evidence. Older adults with hip fractures frequently present with baseline frailty and muscle loss. Following trauma, acute inflammation induces capillary leakage and serum albumin redistribution, resulting in hypoproteinemia (107). Concurrently, malnutrition compromises intestinal barrier integrity and nutrient absorption (108), creating a vicious cycle of inflammation and malnutrition that heightens infection risk (109). Clinically, these composite indices capture this dual state of depleted protein reserves and impaired immune defense. Furthermore, this persistent inflammatory burden not only depletes proteins but also disrupts hematopoiesis. Under such conditions, altered hepatic synthesis and iron sequestration impair erythropoiesis (110), leading to declining hemoglobin levels and widened red blood cell distribution width (111), which are reflected by changes in HRR, RAR, HALP, and CONUT. Although quantitative synthesis was precluded, narrative synthesis suggests these specific hematological indicators may also serve as potential prognostic indicators of adverse outcomes.

Moreover, our findings indicate that GAR may hold prognostic value for short-term complications, such as postoperative pneumonia (102), urinary tract infections (103), and delirium (77). This index evaluates the superimposed effects of trauma-induced acute insulin resistance and nutritional depletion. This combined energy and nutritional deficit reduces the physiological compensatory capacity to withstand surgical stress, thereby precipitating early postoperative complications. Reinforcing the critical role of preoperative reserves, subgroup analyses using GNRI as a baseline nutritional indicator revealed that varying degrees of baseline nutritional depletion may also influence prognostic risk estimates. Furthermore, the prognostic utility of these nutritional indices extends beyond hip fractures. Recent evidence highlights that both GNRI and PNI are valuable prognostic indicators for the broader population of geriatric orthopedic trauma patients requiring intensive care (112). Consequently, these metabolic and nutritional indices may serve as adjunctive tools to help clinicians identify patients at elevated risk, thereby supporting personalized perioperative management and timely interventions. Specifically, identifying high-risk patients should prompt proactive clinical steps, such as early nutritional optimization for those with metabolic depletion, alongside stringent infection prophylaxis and structured postoperative rehabilitation to mitigate these associated adverse outcomes.

## 5 Limitations

Several methodological limitations must be acknowledged. First, based on the QUIPS assessment, some effect estimates relied on crude ORs due to raw data limitations. This lack of adjustment for confounders introduces a risk of bias and could potentially inflate the pooled effects. Second, publication bias was detected in certain indicators. Therefore, these specific findings should be interpreted with caution. Third, although effect directions across different measurement time points were consistent, this temporal confounding inevitably reduced the precision of the pooled effect sizes. Fourth, as highlighted by the variability in biomarker cut-offs across included studies, the lack of standardization limits the feasibility of establishing universally applicable clinical intervention thresholds. Fifth, the predominance of retrospective studies limits the establishment of definitive causal relationships and introduces inherent selection bias. Sixth, the geographic concentration of the included data may limit the generalizability of our findings to broader global populations. Therefore, future prospective, large-scale, multicenter cohort studies across diverse populations are required to establish standardized diagnostic thresholds and optimal measurement timing.

## 6 Conclusion

The present investigation validates the prognostic value of pathomechanism-based composite biomarkers among the geriatric population presenting with hip fractures. Specifically, indices reflecting the systemic immune-inflammatory, inflammatory-nutritional, and metabolic-nutritional dimensions serve as robust tools for evaluating the risk of adverse postoperative outcomes. In particular, categorical variables defined by cutoff values have demonstrated more robust prognostic utility and yielded higher-certainty evidence compared to continuous variables. These indicators are derived from routine blood tests. Consequently, they can assist clinicians in rapidly and easily identifying vulnerable patients at high risk for postoperative complications and mortality.

## Data Availability

The original contributions presented in the study are included in the article/Supplementary material, further inquiries can be directed to the corresponding authors.

## Footnotes

1
https://www.crd.york.ac.uk/PROSPERO/

## Supplementary material

The Supplementary material for this article can be found online at: https://www.frontiersin.org/articles/10.3389/fnut.2026.1881336/full#supplementary-material. References (113–115) are cited in the supplementary materials.

## References

[1] Zhang X Xu D Yang Y Zeng Y Yu H Fang T . Intrinsic capacity and risk of hip fracture in older adults: evidence from five multinational aging cohorts. J Nutr Health Aging. (2026) 30:100773. doi: 10.1016/j.jnha.2026.100773, 41520477 PMC12819345

[2] Han X Han L Chu F Liu B Song F Jia D . Predictors for 1-year mortality in geriatric patients following fragile intertrochanteric fracture surgery. J Orthop Surg Res. (2024) 19:701. doi: 10.1186/s13018-024-05219-4, 39472932 PMC11523668

[3] Bui M Nijmeijer WS Hegeman JH Witteveen A Groothuis-Oudshoorn CGM. Systematic review and Meta-analysis of preoperative predictors for early mortality following hip fracture surgery. Osteoporos Int. (2024) 35:561–74. doi: 10.1007/s00198-023-06942-0, 37996546 PMC10957669

[4] Tian P Yang Y He T Wang L Zhang Q Cai Y. Impact of frailty on postoperative complications in older adults after hip fracture: a systematic review of observational studies. Front Med. (2025) 12:1667462. doi: 10.3389/fmed.2025.1667462, 41368319 PMC12682881

[5] Warren M Bretherton C Parker M. Delay to surgery beyond 12 hours is associated with increased hip fracture mortality. Eur J Orthop Surg Traumatol. (2024) 34:2973–80. doi: 10.1007/s00590-024-03997-5, 38844565 PMC11377486

[6] Guzmán-Muñoz E Concha-Cisternas Y Vásquez-Muñoz M Yañez-Sepúlveda R Núñez-Espinosa C Saporte SB . In-hospital mortality among 40,253 older adults with hip fracture: survival outcomes and multivariate analysis in a Chilean cohort. J Clin Med. (2025) 14:7717. doi: 10.3390/jcm14217717, 41227113 PMC12608696

[7] Goh EL Khatri A Costa AB Ting A Steiner K Png ME . Prevalence of complications in older adults after hip fracture surgery: a systematic review and meta-analysis. The Bone Joint J. (2025) 107-B:139–48. doi: 10.1302/0301-620X.107B2.BJJ-2024-0251.R1, 39889748

[8] Guzmán-Muñoz E Vásquez-Muñoz M Concha-Cisternas Y Olivares-Ordenes R Clemente-Suárez V Castillo-Paredes A . Supervised machine learning-based prediction of in-hospital mortality following hip fracture in older adults. Diagnostics. (2026) 16:612. doi: 10.3390/diagnostics16040612, 41750760 PMC12938910

[9] Masionis P Vaitukaitis G Masionienė A Uvarovas V Šatkauskas I. Risk factors associated with complications and early mortality of hip fracture surgery in elderly patients. Medicina. (2026) 62:825. doi: 10.3390/medicina62050825, 42195078 PMC13208852

[10] Downey C Kelly M Quinlan JF. Changing trends in the mortality rate at 1-year post hip fracture - a systematic review. World J Orthop. (2019) 10:166–75. doi: 10.5312/wjo.v10.i3.166, 30918799 PMC6428998

[11] Xi S Wu Z Cui J Yin S Xi S Liu C. Association between frailty, as measured by the frail scale, and 1-year mortality in older patients undergoing hip fracture surgery. BMC Geriatr. (2025) 25:65. doi: 10.1186/s12877-025-05716-z, 39885410 PMC11780830

[12] Andaloro S Cacciatore S Risoli A Comodo RM Brancaccio V Calvani R . Hip fracture as a systemic disease in older adults: a narrative review on multisystem implications and management. Med Sci. (2025) 13:89. doi: 10.3390/medsci13030089, 40700118 PMC12285999

[13] Meermans G van Egmond JC. Malnutrition in older hip fracture patients: prevalence, pathophysiology, clinical outcomes, and treatment—a systematic review. J Clin Med. (2025) 14:5662. doi: 10.3390/jcm14165662, 40869488 PMC12386537

[14] Su Y-H Huang Y-W Kuo Y-J Chuang T-Y Huang S-W Chen Y-P. Prognostic value of three inflammatory markers for mortality after hip fracture in the geriatric population. Sci Rep. (2025) 15:45348. doi: 10.1038/s41598-025-29665-0, 41291019 PMC12749701

[15] Zhu Y Ao T Li F. Association between C-reactive protein-to-lymphocyte ratio and depression: a cross-sectional study. Medicine. (2026) 105:e47563. doi: 10.1097/MD.0000000000047563, 41650030 PMC12885703

[16] Mi L Lakhani I Lee S Wong WT Tse G Fang F. C-reactive protein-to-albumin ratio as a novel prognostic biomarker for long-term mortality in pericarditis: a real-world study. BMC Cardiovasc Disord. (2025) 26:100. doi: 10.1186/s12872-025-05464-3, 41462096 PMC12866473

[17] Wier J Duong AM Jones IA Telang S Heckmann ND Patterson JT. Neutrophil-lymphocyte ratios and optimal surgical timing for hemiarthroplasty for femoral neck fracture. J Am Acad Orthop Surg. (2026) 34:e447–56. doi: 10.5435/JAAOS-D-24-01357, 40668681

[18] Liu C Zheng J Bai Y Gan L Gu Y. Neutrophil-to-lymphocyte ratio for predicting postoperative mortality after hip fracture surgery: a systematic review and Meta-analysis. J Orthop Surg Res. (2025) 20:1072. doi: 10.1186/s13018-025-06495-4, 41402900 PMC12706907

[19] Pan W-G Chou Y-C Wu J-L Yeh T-T. Impact of hematologic inflammatory markers on the prognosis of geriatric hip fracture: a systematic review and Meta-analysis. Eur J Med Res. (2024) 29:609. doi: 10.1186/s40001-024-02211-w, 39702270 PMC11657849

[20] Liu N Lv L Jiao J Zhang Y Zuo X-L. Association between nutritional indices and mortality after hip fracture: a systematic review and Meta-analysis. Eur Rev Med Pharmacol Sci. (2023) 27:2297–304. doi: 10.26355/eurrev_202303_31763, 37013747

[21] Page MJ McKenzie JE Bossuyt PM Boutron I Hoffmann TC Mulrow CD . The Prisma 2020 statement: an updated guideline for reporting systematic reviews. BMJ. (2021) 372:n71. doi: 10.1136/bmj.n71, 33782057 PMC8005924

[22] Borenstein M Hedges LV Higgins JPT Rothstein HR. "Effect sizes based on binary data (2×2 tables)". In: Introduction to Meta-Analysis. Chichester: John Wiley & Sons, Ltd (2009). p. 33–9. doi: 10.1002/9780470743386

[23] VanderWeele TJ. Optimal approximate conversions of odds ratios and Hazard ratios to risk ratios. Biometrics. (2020) 76:746–52. doi: 10.1111/biom.13197, 31808145

[24] Hayden JA van der Windt DA Cartwright JL Côté P Bombardier C. Assessing Bias in studies of prognostic factors. Ann Intern Med. (2013) 158:280–6. doi: 10.7326/0003-4819-158-4-201302190-00009, 23420236

[25] DerSimonian R Laird N. Meta-analysis in clinical trials. Control Clin Trials. (1986) 7:177–88. doi: 10.1016/0197-2456(86)90046-2, 3802833

[26] Higgins JPT Thompson SG. Quantifying heterogeneity in a Meta-analysis. Stat Med. (2002) 21:1539–58. doi: 10.1002/sim.1186, 12111919

[27] Knapp G Hartung J. Improved tests for a random effects Meta-regression with a single covariate. Stat Med. (2003) 22:2693–710. doi: 10.1002/sim.1482, 12939780

[28] Harrer M Cuijpers P Furukawa T Ebert D. Doing Meta-Analysis with R: A Hands-on Guide. New York, NY: Chapman and Hall/CRC (2021). doi: 10.1201/9781003107347

[29] Röver C Knapp G Friede T. Hartung-Knapp-Sidik-Jonkman approach and its modification for random-effects Meta-analysis with few studies. BMC Med Res Methodol. (2015) 15:99–105. doi: 10.1186/s12874-015-0091-1, 26573817 PMC4647507

[30] Duval S Tweedie R. A nonparametric “trim and fill” method of accounting for publication bias in meta-analysis. J Am Stat Assoc. (2000) 95:89–98. doi: 10.1080/01621459.2000.10473905

[31] Higgins JPT Thomas J Chandler J Cumpston M Li T Page MJ . Cochrane Handbook for Systematic Reviews of Interventions. Version 6.4 ed. Chichester: Wiley (2023). doi: 10.1002/9781119536604

[32] Begg CB Mazumdar M. Operating characteristics of a rank correlation test for publication Bias. Biometrics. (1994) 50:1088–101. doi: 10.2307/2533446, 7786990

[33] Egger M Davey Smith G Schneider M Minder C. Bias in meta-analysis detected by a simple, graphical test. BMJ. (1997) 315:629–34. doi: 10.1136/bmj.315.7109.629 9310563 PMC2127453

[34] Viechtbauer W. Conducting meta-analyses in R with the Metafor package. J Stat Softw. (2010) 36:1–48. doi: 10.18637/jss.v036.i03

[35] Bürkner P-C. Brms: an R package for Bayesian multilevel models using Stan. J Stat Softw. (2017) 80:1–28. doi: 10.18637/jss.v080.i01

[36] Wickham H. Ggplot2: Elegant Graphics for Data Analysis. 2nd ed. Cham: Springer International Publishing (2016).

[37] McGuinness LA Higgins JPT. Risk-of-Bias visualization (Robvis): an R package and shiny web app for visualizing risk-of-Bias assessments. Res Synth Methods. (2021) 12:55–61. doi: 10.1002/jrsm.1411, 32336025

[38] Huguet A Hayden JA Stinson J McGrath PJ Chambers CT Tougas ME . Judging the quality of evidence in reviews of prognostic factor research: adapting the Grade framework. Syst Rev. (2013) 2:71. doi: 10.1186/2046-4053-2-71, 24007720 PMC3930077

[39] Ren H Wu L Hu W Ye X Yu B. Prognostic value of the C-reactive protein/prognostic nutritional index ratio after hip fracture surgery in the elderly population. Oncotarget. (2017) 8:61365–72. doi: 10.18632/oncotarget.18135, 28977869 PMC5617429

[40] Temiz A Ersözlü S. Admission neutrophil-to-lymphocyte ratio and postoperative mortality in elderly patients with hip fracture. Ulus Travma Acil Cerrahi Derg. (2019) 25:71–4. doi: 10.5505/tjtes.2018.94572, 30742290

[41] Turgut N Ünal AM. Standard and newly defined prognostic factors affecting early mortality after hip fractures. Cureus. (2022) 14:e21464. doi: 10.7759/cureus.21464, 35223248 PMC8860722

[42] Tunçez M Bulut T Süner U Önder Y Kazımoğlu C. Prognostic nutritional index (Pni) is an independent risk factor for the postoperative mortality in geriatric patients undergoing hip arthroplasty for femoral neck fracture? A prospective controlled study. Arch Orthop Trauma Surg. (2024) 144:1289–95. doi: 10.1007/s00402-024-05201-z, 38265465

[43] Fujimoto Y Setoguchi T Ishidou Y Taniguchi N. Low geriatric nutritional risk index is a risk factor for death within 1 year following hip fracture. J Orthop Surg. (2022) 30:10225536221103360. doi: 10.1177/10225536221103360, 35578747

[44] Wu Q Ding W Zhang Z La R You D Ding Q . Association between prognostic nutritional index and risk of moderate-to-severe basic activities of daily living dependence two years after hip fracture surgery in older adults. BMC Geriatr. (2025) 25:634–46. doi: 10.1186/s12877-025-06278-w, 40826387 PMC12360021

[45] Wu W Zhu H Chen X Gao Y Tian C Rui C . Geriatric nutritional risk index and prognostic nutritional index as predictors of one-year mortality in older patients after hip fracture surgery: a retrospective cohort study. Geriatr Orthop Surg Rehabil. (2025) 16:21514593251340568. doi: 10.1177/21514593251340568, 40352433 PMC12064898

[46] Xu Y Luo Y. Preoperative prognostic nutritional index is a predictive factor for postoperative delirium in elderly patients with femoral neck fracture. Clin Interv Aging. (2025) 20:941–50. doi: 10.2147/CIA.S518366, 40621090 PMC12229154

[47] Xing H Xiang D Li Y Ji X Xie G. Preoperative prognostic nutritional index predicts postoperative delirium in elderly patients after hip fracture surgery. Psychogeriatrics. (2020) 20:487–94. doi: 10.1111/psyg.12511, 31951677

[48] Mi X Jia Y Song Y Liu K Liu T Han D . Preoperative prognostic nutritional index value as a predictive factor for postoperative delirium in older adult patients with hip fractures: a secondary analysis. BMC Geriatr. (2024) 24:21. doi: 10.1186/s12877-023-04629-z, 38178002 PMC10768121

[49] Chen Y Liu G Zhang J Ge Y Tan Z Peng W . Prognostic nutritional index (Pni) as an independent predictor of 3-year postoperative mortality in elderly patients with hip fracture: a post hoc analysis of a prospective cohort study. Orthop Surg. (2024) 16:2761–70. doi: 10.1111/os.14200, 39142664 PMC11541133

[50] Chen Y Bei M Liu G Zhang J Ge Y Tan Z . Prognostic nutritional index (Pni) is an independent predictor for functional outcome after hip fracture in the elderly: a prospective cohort study. Arch Osteoporos. (2024) 19:107. doi: 10.1007/s11657-024-01469-1, 39499371 PMC11538184

[51] Arslan K Celik S Arslan HC Sahin AS Genc Y Erturk C. Predictive value of prognostic nutritional index on postoperative intensive care requirement and mortality in geriatric hip fracture patients. North Clin Istanb. (2024) 11:249–57. doi: 10.14744/nci.2024.60430, 39005743 PMC11237825

[52] Yotsuya K Yamazaki K Sarukawa J Yasuda T Matsuyama Y. Relationship between the perioperative prognostic nutritional index and postoperative gait function in elderly hip fractures. Osteoporos Sarcopenia. (2024) 10:72–7. doi: 10.1016/j.afos.2024.05.006, 39035227 PMC11260016

[53] Hong SW Jeong HC Kim SH. The neutrophil-to-lymphocyte ratio and preoperative pulmonary function test results as predictors of in-hospital postoperative complications after hip fracture surgery in older adults. J Clin Med. (2023) 12:108. doi: 10.3390/jcm12010108, 36614909 PMC9821284

[54] Faust LM Lerchenberger M Gleich J Linhart C Keppler AM Schmidmaier R . Predictive value of prognostic nutritional index for early postoperative mobility in elderly patients with Pertrochanteric fracture treated with intramedullary nail Osteosynthesis. J Clin Med. (2023) 12:1792. doi: 10.3390/jcm12051792, 36902579 PMC10003114

[55] Popp D Stich-Regner M Schmoelz L Silvaieh S Heisinger S Nia A. Predictive feasibility of the Graz malnutrition screening, controlling nutritional status score, geriatric nutritional risk index, and prognostic nutritional index for postoperative Long-term mortality after surgically treated proximal femur fracture. Nutrients. (2024) 16:4280. doi: 10.3390/nu16244280, 39770903 PMC11676286

[56] Long A Yang D Jin L Zhao F Wang X Zhang Y . Admission inflammation markers influence Long-term mortality in elderly patients undergoing hip fracture surgery: a retrospective cohort study. Orthop Surg. (2024) 16:38–46. doi: 10.1111/os.13932, 37984859 PMC10782247

[57] Aydın A Kaçmaz O. Crp/albumin ratio in predicting 1-year mortality in elderly patients undergoing hip fracture surgery. Eur Rev Med Pharmacol Sci. (2023) 27:8438–46. doi: 10.26355/eurrev_202309_33770, 37782161

[58] Capkin S Guler S Ozmanevra R. C-reactive protein to albumin ratio may predict mortality for elderly population who undergo hemiarthroplasty due to hip fracture. J Investig Surg. (2021) 34:1272–7. doi: 10.1080/08941939.2020.1793038, 32668996

[59] Balta O Altınayak H Gürler Balta M Astan S Uçar C Kurnaz R . Can C-reactive protein-based biomarkers be used as predictive of 30-day mortality in elderly hip fractures?A retrospective study. Ulus Travma Acil Cerrahi Derg. (2022) 28:849–56. doi: 10.14744/tjtes.2022.12454, 35652864 PMC10443014

[60] Vural A Dolanbay T Yagar H. Hemoglobin, albumin, lymphocyte and platelet (Halp) score for predicting early and late mortality in elderly patients with proximal femur fractures. PLoS One. (2025) 20:e0313842. doi: 10.1371/journal.pone.0313842, 39787124 PMC11717259

[61] Cacciola G Mancino F Holzer LA De Meo F De Martino I Bruschetta A . Predictive value of the C-reactive protein to albumin ratio in 30-day mortality after hip fracture in elderly population: a retrospective observational cohort study. J Clin Med. (2023) 12:4544. doi: 10.3390/jcm12134544, 37445579 PMC10342779

[62] Kim HJ Lee S Kim SH Lee S Sim JH Ro YJ. Association of C-reactive protein to albumin ratio with postoperative delirium and mortality in elderly patients undergoing hip fracture surgery: a retrospective cohort study in a single large center. Exp Gerontol. (2023) 172:112068. doi: 10.1016/j.exger.2022.112068, 36549547

[63] Wang Z Liu H Liu M. The hemoglobin, albumin, lymphocyte, and platelet score as a useful predictor for mortality in older patients with hip fracture. Front Med. (2025) 12:1450818. doi: 10.3389/fmed.2025.1450818, 40041469 PMC11876120

[64] Tahak F Yaka H Kirilmaz A Kekeç AF Çolak TS Özer M. Relationship between mortality and halp score in femoral neck fractures treated with hemiarthroplasty. Jt Dis Relat Surg. (2025) 36:589–95. doi: 10.52312/jdrs.2025.2093 40783990 PMC12456328

[65] Choi YH Wong PY Fong MK. A potential novel predictor of 1-year mortality in elderly hip fracture patients-albumin Haemoglobin index. Osteoporosis Int. (2025) 36:1557–64. doi: 10.1007/s00198-025-07566-2, 40493243 PMC12460436

[66] Coşkun Yaş S Yaş S Ayhan B Atalay İ. Prognostic value of the red cell distribution width to albumin ratio in predicting mortality in elderly hip fracture patients undergoing hemiarthroplasty. Eur J Trauma Emerg Surg. (2025) 51:314. doi: 10.1007/s00068-025-02995-4, 41148332

[67] Cao X Zhang H Li Y Liu M He J Li J. Hemoglobin to red cell distribution width ratio predicts postoperative delirium in older patients undergoing hip fracture surgery: a retrospective cohort study. Geriatr Gerontol Int. (2025) 25:565–71. doi: 10.1111/ggi.70023, 40047152

[68] Yuan X Zeng W Wang H Shu G Wu C Nie M . Predictive value of the early postoperative hemoglobin-to-red blood cell distribution width ratio for acute kidney injury in elderly intertrochanteric fracture patients. BMC Musculoskelet Disord. (2024) 25:630. doi: 10.1186/s12891-024-07745-y, 39113005 PMC11308471

[69] Cheng XQ Chen W Yan JC Yang ZB Li CS Wu DW . Association of Preoperative Nutritional Status Evaluated by the controlling nutritional status score with walking Independence at 180 days postoperatively: a prospective cohort study in Chinese older patients with hip fracture. Int J Surg. (2023) 109:2660–71. doi: 10.1097/js9.0000000000000497, 37226868 PMC10498878

[70] Chen Y Guo Y Tong G He Y Zhang R Liu Q. Combined nutritional status and activities of daily living disability is associated with one-year mortality after hip fracture surgery for geriatric patients: a retrospective cohort study. Aging Clin Exp Res. (2024) 36:127. doi: 10.1007/s40520-024-02786-8, 38849714 PMC11161424

[71] Chen X Fan Y Tu H Chen J. A novel nomogram developed based on preoperative immune inflammation-related indicators for the prediction of postoperative delirium risk in elderly hip fracture cases: a single-center retrospective cohort study. J Inflamm Res. (2024) 17:7155–69. doi: 10.2147/JIR.S485181, 39398226 PMC11471118

[72] Chen Y Tu C Liu G Peng W Zhang J Ge Y . Association between admission inflammatory indicators and 3-year mortality risk in geriatric patients after hip fracture surgery: a retrospective cohort study. Front Surg. (2024) 11:1440990. doi: 10.3389/fsurg.2024.1440990, 39229251 PMC11368716

[73] He R Wang F Shen H Zeng Y Zhang L. Association between increased neutrophil-to-lymphocyte ratio and postoperative delirium in elderly patients with total hip arthroplasty for hip fracture. BMC Psychiatry. (2020) 20:496. doi: 10.1186/s12888-020-02908-2 33028273 PMC7539448

[74] Li F Fu X Ruan Y Yu J Chen J Xu L . Association of Inflammatory Markers and Surgical Intervention with postoperative pneumonia in patients with femoral intertrochanteric fracture: a propensity score-matched cohort study. J Orthop. (2025) 67:326–34. doi: 10.1016/j.jor.2025.06.025, 40741072 PMC12304692

[75] Liu XY Guo SQ Chen XM Zeng WN Zhou ZK. Correlation of preoperative inflammation/immunity markers with postoperative urinary tract infections in elderly hip fracture patients. Orthop Surg. (2025) 17:2350–61. doi: 10.1111/os.70107, 40637046 PMC12318684

[76] Zhou J Fu J Zhao Q Lin S Zhu H. Effect of neutrophil-to-lymphocyte ratio on short-term prognosis of elderly patients with hip fracture. Am J Transl Res. (2021) 13:9122–8. 34540026 PMC8430054

[77] Wang W Yao W Tang W Li Y Liu Y Lv Q . Glucose-to-albumin ratio as a new predictive Indicator for postoperative delirium in geriatric hip fracture patients. J Arthroplast. (2025) 40:1573–81.e4. doi: 10.1016/j.arth.2024.11.037, 39608679

[78] Zhao S Sun T Zhang J Wang Y Guo Y Wang X. How to predict postoperative delirium in geriatric patients with hip fracture as soon as possible? A retrospective study. BMC Surg. (2024) 24:306. doi: 10.1186/s12893-024-02599-6, 39395962 PMC11470640

[79] Yao W Wang W Tang W Lv Q Ding W. Neutrophil-to-lymphocyte ratio (Nlr), platelet-to-lymphocyte ratio (Plr), and systemic immune inflammation index (Sii) to predict postoperative pneumonia in elderly hip fracture patients. J Orthop Surg Res. (2023) 18:673. doi: 10.1186/s13018-023-04157-x, 37697317 PMC10496383

[80] Ji S Li Z Li M Lu Y Ma T Qi H . Relationship between neutrophil/lymphocyte ratio and postoperative delirium in elderly patients with proximal femoral nail anti-rotation for intertrochanteric fractures. Am J Transl Res. (2023) 15:1367–73. 36915754 PMC10006771

[81] Ding K Shang Z Sun D Yang W Zhang Y Wang L . The admission inflammatory biomarkers profile of elderly hip fractures and its association with one-year walking Independence and mortality: a prospective study. Int Orthop. (2025) 49:19–28. doi: 10.1007/s00264-024-06353-8, 39466411

[82] Bala MM. The benefit of dynamic neutrophil-lymphocyte ratio and systemic immune-inflammation index in predicting survival in patients undergoing hemiarthroplasty. Eur Rev Med Pharmacol Sci. (2022) 26:3878–85. doi: 10.26355/eurrev_202206_28955, 35731057

[83] Bingol O Ozdemir G Kulakoglu B Keskin OH Korkmaz I Kilic E. Admission neutrophil-to-lymphocyte ratio and monocyte-to-lymphocyte ratio to predict 30-day and 1-year mortality in geriatric hip fractures. Injury. (2020) 51:2663–7. doi: 10.1016/j.injury.2020.07.048, 32739153

[84] Celiksoz A Kavak M Tarlacık AO. Inflammatory index as a predictor of mortality in elderly patients with Intracapsular femoral neck fracture. Cureus. (2023) 15:e46318. doi: 10.7759/cureus.46318, 37790871 PMC10544652

[85] Kekeç AF Çolak TS. Is there a predictive value of the preoperative neutrophil-Lymnhocyte ratio in terms of intensive care need in geriatric patients who underwent Pertrochanteric fracture surgery? Ulus Travma Acil Cerrahi Derg. (2022) 28:1164–9. doi: 10.14744/tjtes.2021.73404, 35920426 PMC10315978

[86] Karadeniz S Yurtbay A. Predicting mortality rate in elderly patients operated for hip fracture using red blood cell distribution width, neutrophil-to-lymphocyte ratio, and Nottingham hip fracture score. Jt Dis Relat Surg. (2022) 33:538–46. doi: 10.52312/jdrs.2022.683, 36345181 PMC9647681

[87] Miniksar ÖH Kaçmaz O. The effect of neutrophil-lymphocyte ratio on admission to postoperative intensive care and mortality in elderly patients undergoing hip fracture surgery with spinal anesthesia. Eur Respir J. (2021) 7:628–34. doi: 10.18621/eurj.835339

[88] Perna A Rovere G Passiatore M Franchini A Macchiarola L Maruccia F . Crystal ball in a blood's drop: unlocking hidden prognostic power in the neutrophil-to-lymphocyte ratio (Nlr) and the platelet-to-lymphocyte ratio (Plr) for elderly hip fracture patients. J Clin Med. (2025) 14:3584. doi: 10.3390/jcm14103584, 40429579 PMC12112460

[89] Golsorkhtabaramiri M Mckenzie J Potter J. Predictability of neutrophil to lymphocyte ratio in preoperative elderly hip fracture patients for post-operative short-term complications: a retrospective study. BMC Musculoskelet Disord. (2023) 24:227. doi: 10.1186/s12891-023-06211-5, 36966301 PMC10039504

[90] Marom O Paz I Segal D Topaz G Abelson N Tavdi A . Proximal femur fractures in the elderly-a novel modality to predict mortality: the neutrophil-to-lymphocyte ratio. J Clin Med. (2023) 12:11. doi: 10.3390/jcm12020456, 36675385 PMC9865272

[91] Kolhe SN Holleyman R Chaplin A Langford S Reed MR Witham MD . Association between markers of inflammation and outcomes after hip fracture surgery: analysis of routinely collected electronic healthcare data. BMC Geriatr. (2025) 25:274. doi: 10.1186/s12877-025-05939-0, 40275223 PMC12023628

[92] Zhou L Huang C Zhu X Ma Z. Combined systemic immune-inflammatory index (Sii) and geriatric nutritional risk index (Gnri) predict survival in elderly patients with hip fractures: a retrospective study. J Orthop Surg Res. (2024) 19:125–35. doi: 10.1186/s13018-024-04585-3, 38321497 PMC10845798

[93] Tan S Jiang Y Qin K Luo Y Liang D Xie Y . Systemic immune-inflammation index and 2-year all-cause mortality in elderly patients with hip fracture. Arch Gerontol Geriatr. (2025) 129:105695. doi: 10.1016/j.archger.2024.105695, 39577025

[94] Wang ZC Jiang W Chen X Yang L Wang H Liu YH. Systemic immune-inflammation index independently predicts poor survival of older adults with hip fracture: a prospective cohort study. BMC Geriatr. (2021) 21:155. doi: 10.1186/s12877-021-02102-3, 33663402 PMC7934427

[95] Çelen ZE. Predictive value of the systemic immune-inflammation index on one-year mortality in geriatric hip fractures. BMC Geriatr. (2024) 24:340. doi: 10.1186/s12877-024-04916-3, 38622572 PMC11020614

[96] Chua JY Yeh KT Lee RP Wu WT. Combining Gnri and Clr index predicts outcome following hip fracture surgery. J Bone Miner Metab. (2025) 44:49–57. doi: 10.1007/s00774-025-01638-3, 41148285

[97] Wang Z Wang H Yang L Jiang W Chen X Liu Y. High platelet-to-lymphocyte ratio predicts poor survival of elderly patients with hip fracture. Int Orthop. (2021) 45:13–21. doi: 10.1007/s00264-020-04833-1, 32989560 PMC7521768

[98] Tekin SB. Relationship between admission neutrophil/lymphocyte, thrombocyte/lymphocyte, and monocyte/lymphocyte ratios and 1-year mortality in geriatric hip fractures: triple comparison. Ulus Travma Acil Cerrahi Derg. (2022) 28:1634–40. doi: 10.14744/tjtes.2021.94799, 36282165 PMC10277357

[99] Yerli M Erkurt N Bayraktar TO Saygılı MS Yüce A Gürbüz H. Can geriatric nutrition risk index predict postoperative 90-day complications and mortality in elderly patients with hip fracture? Eur J Geriatr Gerontol. (2024) 6:8–11. doi: 10.4274/ejgg.galenos.2023.2023-5-2

[100] Funahashi H Morita D Iwase T Asamoto T. Usefulness of nutritional assessment using geriatric nutritional risk index as an independent predictor of 30-day mortality after hip fracture surgery. Orthop Traumatol Surg Res. (2022) 108:103327. doi: 10.1016/j.otsr.2022.103327, 35577274

[101] Tsutsui T Fujiwara T Matsumoto Y Kimura A Kanahori M Arisumi S . Geriatric nutritional risk index as the prognostic factor in older patients with fragility hip fractures. Osteoporos Int. (2023) 34:1207–21. doi: 10.1007/s00198-023-06753-3, 37067545

[102] Tang W Ni X Yao W Wang W Li Y Lv Q . Glucose-albumin ratio (gar) as a novel biomarker for predicting postoperative pneumonia (pop) in older adults with hip fractures. Sci Rep. (2024) 14:26637. doi: 10.1038/s41598-024-60390-2, 39496632 PMC11535218

[103] Wang W Tang W Yao W Lv Q Ding W. Glucose-albumin ratio (gar) as a novel biomarker of postoperative urinary tract infection in elderly hip fracture patients. Front Med. (2024) 11:1366012. doi: 10.3389/fmed.2024.1366012, 39076765 PMC11284060

[104] Liu Z-J Li G-H Wang J-X Mo Z-H Yang K-Y Shen C-L . Prognostic value of the systemic immune-inflammation index in critically ill elderly patients with hip fracture: evidence from mimic (2008–2019). Front Med. (2024) 11:1408371. doi: 10.3389/fmed.2024.1408371, 38873200 PMC11169710

[105] Wang Z Wang L Ji M Lou J Yuan Y Cui Y . Mitochondrial dysfunction in immune cells during the perioperative period: mechanisms, emerging therapeutic strategies, and implications for multi-organ protection. Front Immunol. (2026) 17:1734880. doi: 10.3389/fimmu.2026.1734880, 41983134 PMC13070836

[106] Pries AF Lotzgeselle V Macke C Liodakis E Neunaber C Gogol M. Systemic inflammatory response following hip fracture in older people: postoperative Crp and Pct dynamics and their association with coagulation and renal function. GeroScience. (2026). doi: 10.1007/s11357-026-02227-6, 41915296

[107] Yu Z Li Y Zhang Y Qiu N Zhu W. Analysis of risk factors for hypoproteinemia after Total hip arthroplasty and construction of a nomogram: a retrospective study. Medicine. (2026) 105:e47345. doi: 10.1097/MD.0000000000047345, 41630248 PMC12863805

[108] Di Vincenzo O Cioffi I Marino M Riso P Del Bo’ C Scalfi L. The interrelationships between malnutrition and intestinal permeability in adults: a systematic review and critical appraisal of current evidence. Nutr Res Rev. (2026) 39:e4. doi: 10.1017/S0954422425100255, 41254964

[109] Golenia A Olejnik P. The interplay between inflammation and malnutrition in acute ischemic stroke: a narrative review. Nutr Rev. (2026):nuag040. doi: 10.1093/nutrit/nuag040, 41954896

[110] Kolarš B Mijatović Jovin V Živanović N Minaković I Gvozdenović N Dickov Kokeza I . Iron deficiency and Iron deficiency Anemia: a comprehensive overview of established and emerging concepts. Pharmaceuticals. (2025) 18:1104. doi: 10.3390/ph18081104, 40872496 PMC12389209

[111] Xu D Wu J Zhu Y Guo J Li Y. Association of red Cell Distribution Width-to-Albumin Ratio with mortality in adults with low muscle mass: a retrospective cohort study. J Int Med Res. (2026) 54:3000605251413087. doi: 10.1177/03000605251413087, 41565625 PMC12833119

[112] Arslan K Şahin AS. Prognostic value of geriatric nutritional risk index and prognostic nutritional index in geriatric orthopedic trauma patients in the surgical intensive care unit. Duzce Med J. (2025) 27:11–6. doi: 10.18678/dtfd.1549923

[113] Harville DA. Maximum likelihood approaches to variance component estimation and to related problems. J Am Stat Assoc. (1977) 72:320–38. doi: 10.1080/01621459.1977.10480998, 37339054

[114] Williams DR Rast P Bürkner P-C. Bayesian meta-analysis with weakly informative prior distributions. Psychol Methods. (2018) 23:941–61. doi: 10.31234/osf.io/7tbrm

[115] Vehtari A Gelman A Simpson D Carpenter B Bürkner P-C. Rank-normalization, folding, and localization: an improved R̂ for assessing convergence of Mcmc. Bayesian Anal. (2021) 16:667–718. doi: 10.1214/20-BA1221

