# Supplementary material for: Association of multidimensional biomarkers of immune, nutritional, inflammatory, and metabolic status with postoperative outcomes in older hip fracture patients: a systematic review and meta-analysis
Source: Front Nutr. 2026 Aug 19;13:1881336. doi: 10.3389/fnut.2026.1881336 (PMC13533684; doi:10.3389/fnut.2026.1881336)
Supplement: Supplementary file 1 [file Supplementary_file_1.docx]

Supplementary Material

1. **The specific search terms for each database	1**
2. **Table S1:** Narrative synthesis of the associations between evaluated composite biomarkers and clinical outcomes	**6**
3. **Risk of Bias Assessment Protocol (QUIPS)	13**
4. **Specification of the Bayesian Hierarchical Model	17**
5. **Pre-defined Quantitative Criteria for GRADE Assessment	18**
6. **Table S2:** Definitions and calculation formulas of the evaluated composite biomarkers	**19**
7. **Figure S1:** Risk of bias assessments for the 65 studies included in the narrative review	**20**
8. **Table S3:** GRADE evidence profile and certainty of evidence for the evaluated composite biomarkers	**21**
9. **Figure S2:** Forest plots summarizing the prognostic value of individual composite biomarkers for postoperative outcomes	**23**
10. **Figure S3:** Sbgroup analyses of the associations between composite biomarkers and adverse outcomes	**26**
11. **Table S4:** PRISMA 2020 checklist	**28**

# 1. The specific search terms for each database

| **PubMed** | | |
| --- | --- | --- |
| #1 | ("Hip Fractures"[Mesh] OR "Femoral Neck Fractures"[Mesh] OR "hip fracture*"[Title/Abstract] OR "femoral neck fracture*"[Title/Abstract] OR "femur neck fracture*"[Title/Abstract] OR "trochanteric fracture*"[Title/Abstract] OR "intertrochanteric fracture*"[Title/Abstract] OR "subtrochanteric fracture*"[Title/Abstract]) | 44,041 |
| #2 | ("Aged"[Mesh] OR "Aged, 80 and over"[Mesh] OR "Frailty"[Mesh] OR aged[Title/Abstract] OR elder*[Title/Abstract] OR geriatric*[Title/Abstract] OR "older adult*"[Title/Abstract] OR "older patient*"[Title/Abstract] OR "frail elderly"[Title/Abstract]) | 4,571,296 |
| #3 | ("Biomarkers"[Mesh] OR "Biomarker*"[Title/Abstract] OR "Lymphocytes"[Mesh] OR "Lymphocyte*"[Title/Abstract] OR "neutrophil to lymphocyte ratio"[Title/Abstract] OR "NLR"[Title/Abstract] OR "platelet to lymphocyte ratio"[Title/Abstract] OR "PLR"[Title/Abstract] OR "Systemic Immune-Inflammation Index"[Title/Abstract] OR "SII"[Title/Abstract] OR "Monocyte to lymphocyte ratio"[Title/Abstract] OR "MLR"[Title/Abstract] OR "C-reactive protein to lymphocyte ratio"[Title/Abstract] OR "CLR"[Title/Abstract] OR "Serum Albumin"[Mesh] OR "Serum Albumin"[Title/Abstract] OR "Nutrition Assessment"[Mesh] OR "Nutrition Assessment"[Title/Abstract] OR "prognostic nutritional index"[Title/Abstract] OR "PNI"[Title/Abstract] OR "glucose to albumin ratio"[Title/Abstract] OR "GAR"[Title/Abstract] OR "C-reactive protein to albumin ratio"[Title/Abstract] OR "CAR"[Title/Abstract] OR "red cell distribution width to albumin ratio"[Title/Abstract] OR "RAR"[Title/Abstract] OR "hemoglobin to red cell distribution width ratio"[Title/Abstract] OR "HRR"[Title/Abstract] OR "geriatric nutritional risk index"[Title/Abstract] OR "GNRI"[Title/Abstract] OR "Hemoglobin, Albumin, Lymphocyte and Platelet score"[Title/Abstract] OR "HALP"[Title/Abstract] OR "Controlling nutritional status score"[Title/Abstract] OR "CONUT"[Title/Abstract]) | 2,172,932 |
| #4 | #1 AND #2 AND #3 | 1,059 |

| **Web of Science** | | |
| --- | --- | --- |
| #1 | TS=("hip fracture*" OR "femoral neck fracture*" OR "femur neck fracture*" OR "trochanteric fracture*" OR "intertrochanteric fracture*" OR "subtrochanteric fracture*") | 45,000 |
| #2 | TS=("aged" OR "elder*" OR "geriatric*" OR "older adult*" OR "older patient*" OR "frail*") | 1,542,880 |
| #3 | TS=("Biomarker*" OR "Lymphocyte*" OR "neutrophil to lymphocyte ratio" OR "NLR" OR "platelet to lymphocyte ratio" OR "PLR" OR "Systemic Immune-Inflammation Index" OR "SII" OR "Monocyte to lymphocyte ratio" OR "MLR" OR "C-reactive protein to lymphocyte ratio" OR "CLR" OR "Serum Albumin" OR "Nutrition* Assessment" OR "prognostic nutritional index" OR "PNI" OR "glucose to albumin ratio" OR "GAR" OR "C-reactive protein to albumin ratio" OR "CAR" OR "red cell distribution width to albumin ratio" OR "RAR" OR "hemoglobin to red cell distribution width ratio" OR "HRR" OR "geriatric nutritional risk index" OR "GNRI" OR "Hemoglobin, Albumin, Lymphocyte and Platelet score" OR "HALP" OR "Controlling nutritional status score" OR "CONUT") | 1,451,068 |
| #4 | #1 AND #2 AND #3 | 847 |

| **Scopus** | | |
| --- | --- | --- |
| #1 | TITLE-ABS-KEY( "hip fracture*" OR "femoral neck fracture*" OR "femur neck fracture*" OR "trochanteric fracture*" OR "intertrochanteric fracture*" OR "subtrochanteric fracture*" ) | 61,408 |
| #2 | TITLE-ABS-KEY( "aged" OR "elder*" OR "geriatric*" OR "older adult*" OR "older patient*" OR "frail*" ) | 7,461,989 |
| #3 | TITLE-ABS-KEY( "Biomarkers" OR "Lymphocytes" OR "neutrophil to lymphocyte ratio" OR "NLR" OR "platelet to lymphocyte ratio" OR "PLR" OR "Systemic Immune-Inflammation Index" OR "SII" OR "Monocyte to lymphocyte ratio" OR "MLR" OR "C-reactive protein to lymphocyte ratio" OR "CLR" OR "Serum Albumin" OR "Nutrition Assessment" OR "prognostic nutritional index" OR "PNI" OR "glucose to albumin ratio" OR "GAR" OR "C-reactive protein to albumin ratio" OR "CAR" OR "red cell distribution width to albumin ratio" OR "RAR" OR "hemoglobin to red cell distribution width ratio" OR "HRR" OR "geriatric nutritional risk index" OR "GNRI" OR "Hemoglobin, Albumin, Lymphocyte and Platelet score" OR "HALP" OR "Controlling nutritional status score" OR "CONUT" ) | 2,528,988 |
| #4 | #1 AND #2 AND #3 | 1,255 |

| **Embase** | | |
| --- | --- | --- |
| #1 | ('hip fracture'/exp OR 'femur neck fracture'/exp OR 'hip fracture*':ti,ab,kw OR 'femoral neck fracture*':ti,ab,kw OR 'femur neck fracture*':ti,ab,kw OR 'trochanteric fracture*':ti,ab,kw OR 'intertrochanteric fracture*':ti,ab,kw OR 'subtrochanteric fracture*':ti,ab,kw) | 73,243 |
| #2 | ('aged'/exp OR 'frail elderly'/exp OR 'aged':ti,ab,kw OR 'elder*':ti,ab,kw OR 'geriatric*':ti,ab,kw OR 'older adult*':ti,ab,kw OR 'older patient*':ti,ab,kw) | 5,641,211 |
| #3 | ('biomarker'/exp OR 'biomarker':ti,ab,kw OR 'lymphocyte'/exp OR 'lymphocyte':ti,ab,kw OR 'neutrophil to lymphocyte ratio':ti,ab,kw OR 'nlr':ti,ab,kw OR 'platelet to lymphocyte ratio':ti,ab,kw OR 'plr':ti,ab,kw OR 'systemic immune inflammation index':ti,ab,kw OR 'sii':ti,ab,kw OR 'monocyte to lymphocyte ratio':ti,ab,kw OR 'mlr':ti,ab,kw OR 'c reactive protein to lymphocyte ratio':ti,ab,kw OR 'clr':ti,ab,kw OR 'serum albumin'/exp OR 'serum albumin':ti,ab,kw OR 'nutritional assessment'/exp OR 'nutritional assessment':ti,ab,kw OR 'prognostic nutritional index':ti,ab,kw OR 'pni':ti,ab,kw OR 'glucose to albumin ratio':ti,ab,kw OR 'gar':ti,ab,kw OR 'c reactive protein to albumin ratio':ti,ab,kw OR 'car':ti,ab,kw OR 'red cell distribution width to albumin ratio':ti,ab,kw OR 'rar':ti,ab,kw OR 'hemoglobin to red cell distribution width ratio':ti,ab,kw OR 'hrr':ti,ab,kw OR 'geriatric nutritional risk index':ti,ab,kw OR 'gnri':ti,ab,kw OR 'hemoglobin, albumin, lymphocyte and platelet score':ti,ab,kw OR 'halp':ti,ab,kw OR 'controlling nutritional status score':ti,ab,kw OR 'conut':ti,ab,kw) | 2,363,745 |
| #4 | #1 AND #2 AND #3 | 1,312 |

| **ClinicalTrials.gov** | |
| --- | --- |
| ("Hip Fractures" OR "Femoral Neck Fractures" OR "Trochanteric Fracture" OR "Intertrochanteric Fracture") AND (aged OR elder* OR geriatric* OR "older adult*" OR "older patient*" OR "frail*") AND (Biomarkers OR Lymphocytes OR "neutrophil to lymphocyte ratio" OR NLR OR "platelet to lymphocyte ratio" OR PLR OR "Systemic Immune-Inflammation Index" OR SII OR "Monocyte to lymphocyte ratio" OR MLR OR "C-reactive protein to lymphocyte ratio" OR CLR OR "Serum Albumin" OR "Nutrition Assessment" OR "prognostic nutritional index" OR PNI OR "glucose to albumin ratio" OR GAR OR "C-reactive protein to albumin ratio" OR CAR OR "red cell distribution width to albumin ratio" OR RAR OR "hemoglobin to red cell distribution width ratio" OR HRR OR "geriatric nutritional risk index" OR GNRI OR "Hemoglobin, Albumin, Lymphocyte and Platelet score" OR HALP OR "Controlling nutritional status score" OR CONUT) | 69 |

| **Cochrane Library** | | |
| --- | --- | --- |
| #1 | [mh "Hip Fractures"] OR [mh "Femoral Neck Fractures"] OR (hip NEXT fracture*):ti,ab,kw OR (femoral NEXT neck NEXT fracture*):ti,ab,kw OR (femur NEXT neck NEXT fracture*):ti,ab,kw OR (trochanteric NEXT fracture*):ti,ab,kw OR (intertrochanteric NEXT fracture*):ti,ab,kw OR (subtrochanteric NEXT fracture*):ti,ab,kw | 6,660 |
| #2 | [mh Aged] OR [mh "Aged, 80 and over"] OR [mh Frailty] OR elder*:ti,ab,kw OR geriatric*:ti,ab,kw OR (older NEXT adult*):ti,ab,kw | 350,220 |
| #3 | [mh Biomarkers] OR [mh Lymphocytes] OR Biomarker*:ti,ab,kw OR Lymphocyte*:ti,ab,kw OR NLR OR (neutrophil NEXT to NEXT lymphocyte NEXT ratio*):ti,ab,kw OR PLR OR (platelet NEXT to NEXT lymphocyte NEXT ratio*):ti,ab,kw OR SII OR (Systemic NEXT Immune-Inflammation NEXT Index):ti,ab,kw OR MLR OR (Monocyte NEXT to NEXT lymphocyte NEXT ratio*):ti,ab,kw | 95,970 |
| #4 | [mh "Serum Albumin"] OR [mh "Nutrition Assessment"] OR PNI OR (prognostic NEXT nutritional NEXT index):ti,ab,kw OR GAR OR (glucose NEXT to NEXT albumin NEXT ratio*):ti,ab,kw OR CAR OR (C-reactive NEXT protein NEXT to NEXT albumin NEXT ratio*):ti,ab,kw OR CLR OR (C-reactive NEXT protein NEXT to NEXT lymphocyte NEXT ratio*):ti,ab,kw | 6,827 |
| #5 | RAR OR (red NEXT cell NEXT distribution NEXT width NEXT to NEXT albumin NEXT ratio*):ti,ab,kw OR HRR OR (hemoglobin NEXT to NEXT red NEXT cell NEXT distribution NEXT width NEXT ratio*):ti,ab,kw OR GNRI OR (geriatric NEXT nutritional NEXT risk NEXT index):ti,ab,kw | 1,150 |
| #6 | HALP OR "Hemoglobin Albumin Lymphocyte and Platelet score":ti,ab,kw OR CONUT OR "Controlling nutritional status score":ti,ab,kw | 38 |
| #7 | #1 AND #2 AND (#3 OR #4 OR #5 OR #6) | 120 |

| **ProQuest** | | |
| --- | --- | --- |
| #1 | TI,AB("Hip Fractures" OR "Femoral Neck Fractures" OR "hip fracture*" OR "femoral neck fracture*" OR "femur neck fracture*" OR "trochanteric fracture*" OR "intertrochanteric fracture*" OR "subtrochanteric fracture*") | 15,516 |
| #2 | TI,AB(Aged OR "Aged, 80 and over" OR Frailty OR aged OR elder* OR geriatric* OR "older adult*" OR "older patient*" OR "frail elderly") | 1,495,612 |
| #3 | TI,AB(Biomarker* OR Lymphocytes OR NLR OR "neutrophil to lymphocyte ratio" OR PLR OR "platelet to lymphocyte ratio" OR SII OR "Systemic Immune-Inflammation Index" OR MLR OR "Monocyte to lymphocyte ratio" OR CLR OR "C-reactive protein to lymphocyte ratio" OR "serum albumin" OR "nutrition assessment" OR PNI OR "prognostic nutritional index" OR GAR OR "glucose to albumin ratio" OR CAR OR "C-reactive protein to albumin ratio" OR RAR OR "red cell distribution width to albumin ratio" OR HRR OR "hemoglobin to red cell distribution width ratio" OR GNRI OR "geriatric nutritional risk index" OR HALP OR "Hemoglobin Albumin Lymphocyte and Platelet score" OR CONUT OR "Controlling nutritional status score") | 4,415,415 |
| #4 | #1 AND #2 AND #3 | 250 |

# 2. Table S1

Table S1. Narrative synthesis of the associations between evaluated composite biomarkers and clinical outcomes.

| **Biomarker** | **Association** | **Outcome Category** | **Specific Outcome** | **Direction of association per study** | | | | **Rel. freq. +** | | |
| --- | --- | --- | --- | --- | --- | --- | --- | --- | --- | --- |
|  |  |  |  | **+** | **0** | **−** | |  | | |
| **Immune and Systemic Inflammatory Indices** | | | | | | | | | | |
| **NLR**  **(Continuous)** | Greater risk of poor short- and medium-to-long-term prognosis amongst those with an elevated NLR, whether analyzed as a continuous or dichotomous  variable. | Short-term | POD |  | 87,102 | |  | | | 0/2 |
|  |  |  | POP | 89,84 |  | |  | | | 2/2 |
|  |  |  | UTIs | 85 |  | |  | | | 1/1 |
|  |  |  | in-hospital complications | 100 |  | |  | | | 1/1 |
|  |  |  | ICU admission |  | 101 | |  | | | 0/1 |
|  |  |  | in-hospital mortality |  | 99 | |  | | | 0/1 |
|  |  |  | 30-day mortality | 103 | 101 | |  | | | 1/2 |
|  |  |  | **Total** |  |  | |  | | **5/10** | |
|  |  | Medium-to-long-term | 1-year walking independence | 109^b^ |  | |  | | | 1/1 |
|  |  |  | 90-day mortality | 108,100,15^b^,51^b^ |  | |  | | | 4/4 |
|  |  |  | 3-year mortality |  | 93 | |  | | | 0/1 |
|  |  |  | **Total** |  |  | |  | | | **4/5** |
| **NLR**  **(Dichotomous)** |  | Short-term | POD | 95,97,90 |  | |  | | | 3/3 |
|  |  |  | ICU admission | 95,97 |  | |  | | | 2/2 |
|  |  |  | 30-day mortality | 93^b^ |  | |  | | | 1/1 |
|  |  |  | **Total** |  |  | |  | | | **6/6** |
|  |  | Medium-to-long-term | 1-year mortality | 96,98,84,93,50,86 |  | |  | | | 6/6 |
|  |  |  | **Total** |  |  | |  | | | **6/6** |
| **SII**  **(Continuous)** | Greater risk of poor medium-to-long-term prognosis amongst those with a high SII when analyzed as a dichotomous variable | Short-term | POP |  | 89,84 | |  | | | 0/2 |
|  |  |  | UTIs | 85 |  | |  | | | 1/1 |
|  |  |  | **Total** |  |  | |  | | | **1/3** |
|  |  | Medium-to-long-term | 1-year walking independence |  | 93^b^ | |  | | | 0/1 |
|  |  |  | 1-year mortality | 104 | 108,106 | |  | | | 1/3 |
|  |  |  | 3-year mortality |  | 93 | |  | | | 0/1 |
|  |  |  | **Total** |  |  | |  | | | **1/4** |
| **SII**  **(Dichotomous)** |  | Medium-to-long-term | 1-year mortality | 105,102 |  | |  | | | 2/2 |
|  |  |  | 2-year mortality | 103 |  | |  | | | 1/1 |
|  |  |  | **Total** |  |  | |  | | | **3/3** |
| **MLR**  **(Continuous)** | Greater risk of poor medium-to-long-term prognosis amongst those with a high MLR when analyzed as a dichotomous variable | Short-term | 30-day mortality |  | 101^b^ | |  | | | 0/1 |
|  |  |  | **Total** |  |  | |  | | | **0/1** |
| **MLR**  **(Dichotomous)** |  | Short-term | 30-day mortality | 93^b^ |  | |  | | | 1/1 |
|  |  |  | **Total** |  |  | |  | | | **1/1** |
|  |  | Medium-to-long-term | 1-year mortality | 58,93,108 |  | |  | | | 3/3 |
|  |  |  | 3-year mortality | 93 |  | |  | | | 1/1 |
|  |  |  | **Total** |  |  | |  | | | **4/4** |
| **PLR**  **(Continuous)** | Continuous PLR is predictive of short-term outcomes, while dichotomous PLR correlates with medium-to-long-term prognosis. | Short-term | POP | 89^b^ |  | |  | | | 1/1 |
|  |  |  | UTIs | 85^b^ |  | |  | | | 1/1 |
|  |  |  | **Total** |  |  | |  | | | **2/2** |
|  |  | Medium-to-long-term | 1-year mortality |  | 3^b^ | |  | | | 0/1 |
|  |  |  | 3-year mortality |  | 93^b^ | |  | | | 0/1 |
|  |  |  | **Total** |  |  | |  | | | **0/2** |
| **PLR**  **(Dichotomous)** |  | Medium-to-long-term | 1-year mortality | 107^b^,98^b^ |  | |  | | | 2/2 |
|  |  |  | **Total** |  |  | |  | | | **2/2** |
| **CLR**  **(Continuous)** | No significant association was observed between CLR and short or medium-to-long-term outcomes. | Short-term | 30-day mortality |  | 69^b^ | |  | | | 0/1 |
|  |  |  | **Total** |  |  | |  | | | **0/1** |
|  |  | Medium-to-long-term | 1-year mortality |  | 106^b^ | |  | | | 0/1 |
|  |  |  | **Total** |  |  | |  | | | **0/1** |
| **Basic Nutritional and Metabolic Indices** | | | | | | | | | | |
| **GNRI**  **(Continuous)** | Greater risk of poor short- and medium-to-long-term prognosis amongst those with a lower GNRI,whether analyzed as a continuous or dichotomous variable. | Medium-to-long-term | 1-year mortality | 53^b^,106^b^ |  | |  | | | 2/2 |
|  |  |  | **Total** |  |  | |  | | | **2/2** |
| **GNRI**  **(Dichotomous)** |  | Short-term | 30-day mortality | 110^b^ |  | |  | | | 1/1 |
|  |  |  | **Total** |  |  | |  | | | **1/1** |
|  |  | Medium-to-long-term | 90-day mortality | 109 |  | |  | | | 1/1 |
|  |  |  | 1-year mortality | 112,102 |  | |  | | | 2/2 |
|  |  |  | 5-year mortality | 111 |  | |  | | | 1/1 |
|  |  |  | **Total** |  |  | |  | | | **4/4** |
| **GAR**  **(Dichotomous)** | Greater risk of poor short-term prognosis amongst those with a high GAR when analyzed as a dichotomous variable | Short-term | POD | 87 |  | |  | | | 1/1 |
|  |  |  | POP | 112 |  | |  | | | 1/1 |
|  |  |  | UTIs | 113 |  | |  | | | 1/1 |
|  |  |  | **Total** |  |  | |  | | | **3/3** |
| **Inflammatory-Nutritional Indices** | | | | | | | | | | |
| **PNI**  **(Continuous)** | Greater risk of poor short- and medium-to-long-term prognosis amongst those with a lower PNI,whether analyzed as a continuous or dichotomous variable. | Short-term | POD | 58 |  | |  | | | 1/1 |
|  |  |  | in-hospital complications | 100 |  | |  | | | 1/1 |
|  |  |  | ICU admission | 61^b^ |  | |  | | | 1/1 |
|  |  |  | in-hospital mortality |  | 61 | |  | | | 0/1 |
|  |  |  | **Total** |  |  | |  | | | **2/3** |
|  |  | Medium-to-long-term | BADL independence  at 2 years | 111^b^ |  | |  | | | 1/1 |
|  |  |  | 1-year mortality | 52^b^,87^b^ |  | |  | | | 2/2 |
|  |  |  | **Total** |  |  | |  | | | **3/3** |
| **PNI**  **(Dichotomous)** |  | Short-term | POD | 56,57 | 62 | |  | | | 2/3 |
|  |  |  | 30-day mortality | 85 |  | |  | | | 1/1 |
|  |  |  | walking dependence at discharge | 84 |  | |  | | | 1/1 |
|  |  |  | **Total** |  |  | |  | | | **4/5** |
|  |  | Medium-to-long-term | 1-year mortality | 43^b^ |  | |  | | |  |
|  |  |  | 3-year mortality | 98^b^ |  | |  | | |  |
|  |  |  | 120-day walking dependence | 60^b^ |  | |  | | |  |
|  |  |  | **Total** |  |  | |  | | | **3/3** |
| **CONUT**  **(Dichotomous)** | Greater risk of poor Medium-to-long-term prognosis amongst those with a high CONUT when analyzed as a dichotomous variable | Medium-to-long-term | 180-day walking dependence | 106^b^ |  | |  | | | 1/1 |
|  |  |  | 1-year mortality | 80^b^ |  | |  | | | 1/1 |
|  |  |  | **Total** |  |  | |  | | | **2/2** |
| **HALP**  **(Dichotomous)** | Greater risk of poor Medium-to-long-term prognosis amongst those with a low HALP when analyzed as a dichotomous variable | Medium-to-long-term | 90-day mortality | 73^b^ |  | |  | | | 1/1 |
|  |  |  | 6-month mortality | 74^b^ |  | |  | | | 1/1 |
|  |  |  | 1-year mortality | 90^b^ |  | |  | | | 1/1 |
|  |  |  | **Total** |  |  | |  | | | **3/3** |
| **RAR**  **(Continuous)** | Greater risk of poor short- and Medium-to-long-term prognosis amongst those with an elevated RAR,whether analyzed as a continuous or dichotomous variable. | Short-term | in-hospital mortality | 106^b^ |  | |  | | | 1/1 |
|  |  |  | **Total** |  |  | |  | | | 1/1 |
|  |  | Medium-to-long-term | 3-month mortality | 106^b^ |  | |  | | | 1/1 |
|  |  |  | 1-year mortality | 106^b^ |  | |  | | | 1/1 |
|  |  |  | **Total** |  |  | |  | | | **1/1** |
| **RAR**  **(Dichotomous)** |  | Medium-to-long-term | 3-month mortality | 75^b^ |  | |  | | | 1/1 |
|  |  |  | 6-month mortality | 75^b^ |  | |  | | | 1/1 |
|  |  |  | 1-year mortality | 75^b^ |  | |  | | | 1/1 |
|  |  |  | **Total** |  |  | |  | | | **1/1** |
| **HRR**  **(Continuous)** | Greater risk of poor short-term prognosis amongst those with a lower HRR when analyzed as a continuous variable | Short-term | POD | 77^b^ |  | |  | | | 1/1 |
|  |  |  | AKI | 80^b^ |  | |  | | | 1/1 |
|  |  |  | **Total** |  |  | |  | | | **2/2** |
| **CAR**  **(Continuous)** | Greater risk of poor short- and Medium-to-long-term prognosis amongst those with an elevated CAR,whether analyzed as a continuous or dichotomous variable. | Short-term | POD | 72 |  | |  | | | 1/1 |
|  |  |  | ICU admission | 101 |  | |  | | | 1/1 |
|  |  |  | 30-day mortality | 69 |  | |  | | | 1/1 |
|  |  |  | **Total** |  |  | |  | | | **3/3** |
|  |  | Medium-to-long-term | 1-year mortality | 2^b^ |  | |  | | | 1/1 |
|  |  |  | **Total** |  |  | |  | | | **1/1** |
| **CAR**  **(Dichotomous)** |  | Short-term | 30-day mortality | 71^b^ |  | |  | | | 1/1 |
|  |  |  | **Total** |  |  | |  | | | **1/1** |
|  |  | Medium-to-long-term | 1-year mortality | 72,90,84 |  | |  | | | 3/3 |
|  |  |  | **Total** |  |  | |  | | | **3/3** |

This table presents a narrative synthesis of the findings from the included studies. The superscript [b] denotes studies that were not included in the quantitative meta-analysis and are presented for narrative synthesis only. The direction of the association is categorized as positive (+), null (0), or negative (−) based on statistical significance. Rel. freq. indicates the relative frequency of the observed association direction across the relevant studies. AKI, Acute kidney injury; BADL, Basic activities of daily living; ICU, Intensive care unit; POD, Postoperative delirium; POP, Postoperative pneumonia; UTI, Urinary tract infection; CAR, C-reactive protein-to-albumin ratio; GAR, glucose-to-albumin ratio; GNRI, geriatric nutritional risk index; MLR, monocyte-to-lymphocyte ratio; NLR, neutrophil-to-lymphocyte ratio; PNI, prognostic nutritional index; SII, systemic immune-inflammation index; CLR, C-reactive protein to lymphocyte ratio; PLR, platelet to lymphocyte ratio; CONUT, controlling nutritional status score; HRR, hemoglobin to red cell distribution width ratio; RAR, red cell distribution width to albumin ratio; HALP, hemoglobin, albumin, lymphocyte and platelet score.

# 3. Risk of Bias Assessment Protocol (QUIPS)

**D1:Study participation**

**Prompts**

1. Were the selection criteria clearly defined?
   - Yes: Inclusion and exclusion criteria were clearly reported.
   - No: Solely a description of study participants (e.g., number and types of participants described, but no further details on the selection process).
2. Was the study population representative of the population of interest?
   - Yes: The study enrolled consecutive surgically treated patients aged ≥60 years with both femoral neck and intertrochanteric fractures, without systematic exclusion of vulnerable populations (e.g., patients with cognitive impairment or institutionalized residents). If the specific fracture types were not explicitly mentioned but the study broadly stated that “all hip fracture patients” were enrolled, it is assumed that both femoral neck and intertrochanteric fractures were represented.
   - No: The study only included a single fracture type (e.g., only intertrochanteric fractures) or was restricted to a specific surgical technique (e.g., patients undergoing Proximal Femoral Nail Antirotation [PFNA] surgery only).
3. Were the baseline characteristics of the study sample described in sufficient detail?
   - Yes: Baseline characteristics were described in summary tables using descriptive statistics (e.g., gender and age distributions).
   - No: The study sample was only described in a general manner, without descriptive statistics.

**Rating scheme**

- Low risk of bias if:
  - All questions were answered with yes.
- Moderate risk of bias if:
  - At most one question was answered with no.
- High risk of bias if:
  - At least two questions were answered with no.

**D2:Attrition**

**Prompts**

1. Was the completeness of follow-up adequate?
   - Low risk: At most 10% of the data was lost.
   - Moderate risk: 10%-20% of the data was lost.OR no statement on loss of follow-up was provided.
   - High risk: Data loss is greater than 20%.
2. Were there no important differences between participants who completed the study and those who did not?
   - Yes: The data appeared to be missing at random.
   - No: Missing data was correlated with relevant patient characteristics.

**Rating scheme**

- Low risk of bias if:
  - Question 1 was low risk, and question 2 was yes.
- High risk of bias if:
  - Question 1 was high risk, and question 2 was not.
- Moderate risk of bias if:
  - All other cases, such as when question 1 is moderate risk, but question 2 is Yes.

**D3:Prognostic Factor Measurement**

**Prompts**

1. Were prognostic factors clearly defined?
   - Yes: The components of the biomarker and its calculation formula (e.g., based on serum laboratory values) were explicitly described. If a specific cut-off value was used, the exact threshold was provided.
   - No: The description of the biomarker or its components was insufficient to permit replication.
2. Were prognostic factors measured or acquired appropriately?
   - Yes: The biomarkers were measured during the perioperative period using serum samples, and the analysis was performed using standard laboratory methods. The timing of blood collection must be consistent across all subjects (e.g., all within 24 hours of admission or all preoperative) to ensure comparability.
   - No: No clear description of the measurement technique or timing was provided

**Rating scheme**

- Low risk of bias if:
  - All questions were answered with yes.
- Moderate risk of bias if:
  - At most one question was answered with no.
- High risk of bias if:
  - Both questions were answered with no.

**D4:Outcome Measurement**

**Prompts**

1. Is the outcome measure clearly and correctly defined?
   - Yes: Outcome measures, such as specific postoperative complications (e.g., delirium, pneumonia, urinary tract infection, acute kidney injury), ICU admission, postoperative mobility, or mortality rates, were explicitly defined.
   - No: No specific description of the outcome measures was provided.
2. Was the outcome measurement obtained validly?
   - Yes: The diagnosis of complications was based on standard clinical criteria or guidelines. Mortality data were retrieved from medical records, official mortality registries, or through telephone follow-up with patients or relatives.
   - No: No specific description of the outcome measurement or ascertainment was provided.

**Rating scheme**

- Low risk of bias if:
  - All questions were answered with yes.
- Moderate risk of bias if:
  - At most one question was answered with no.
- High risk of bias if:
  - Both questions were answered with no.

**D5:Confounding**

**Prompts and rating**

1. Were the confounding factors controlled?
   - Low Risk: The study adjusted for confounders using at least one multivariable method, such as logistic regression, Cox proportional hazards regression, or propensity score matching (PSM).
   - Moderate risk: Multivariable analysis was performed, but specific confounding factors were not clearly stated (e.g., the study mentioned "adjusted for multiple factors" without listing them).
   - High risk: No multivariable adjustment was performed.

**D6:Analysis and Reporting**

**Prompts**

2. Is the statistical model appropriate for the study design?
   - Yes: Appropriate regression models were used to analyse the influence of prognostic factors in cohort studies (e.g., logistic regression or Cox proportional hazard regression).
   - No: None of the aforementioned regression models were used in the statistical analysis.
3. Were results reported without selective biases?
   - Yes: Risk measures of all covariates included in the multivariate regression models were reported.
   - No: Risk measures of covariates included in multivariate models were suppressed, based on statistical insignificance.

**Rating scheme**

- Low risk of bias if:
  - All questions were answered with yes.
- Moderate risk of bias if:
  - At most one question was answered with no.
- High risk of bias if:
  - Both questions were answered with no.

**Overall Risk of Bias Assessment**

**Prompts**

1. The overall risk of bias of a study was rated as low if:
   - All domains that are bias domains were rated low risk.
   - At most one domain was rated moderate risk.
2. The overall risk of bias of a study is rated as moderate if:
   - At least two domains are rated moderate risk, and all other domains are rated low risk.
3. The overall risk of bias of a study is rated high if:
   - At least one domain was rated to be at high risk of bias.
   - At least four domains were rated to be at moderate risk of bias.

# 4. Specification of the Bayesian Hierarchical Model

**1. Rationale for Bayesian Approach:**

Standard frequentist meta-analysis methods, such as the moment-based estimator proposed by DerSimonian and Laird [25] or the Restricted Maximum Likelihood (REML) estimator [34,113], often underestimate the between-study heterogeneity ($\tau^{2}$) when the number of included studies is limited.In extreme cases,heterogeneity may be erroneously estimated as zero, leading to artificially narrow confidence intervals and potential false-positive results. To address this limitation and rigorously test the robustness of our primary findings, we performed sensitivity analyses using a Bayesian hierarchical model with weakly informative priors, following the methodological framework described by Bui et al. [5] and Harrer et al. [28].

**2. Model Specification:**

Let $\theta_{i}$ be the observed effect size (e.g., log odds ratio) for study $i$ and $\sigma_{i}^{2}$ be the within-study variance. The hierarchical model assumes that the true effect sizes across studies follow a normal distribution:

$$\theta_{i}\sim N\left( \mu,\tau^{2} \right)$$

**Where:**

- $\mu$ represents the pooled (mean) effect size.
- $\tau$ represents the between-study standard deviation (heterogeneity).

**3. Prior Distributions:**

To prevent the underestimation of heterogeneity while avoiding the introduction of strong subjective bias, we specified the following weakly informative priors based on recommendations for small-sample meta-analyses [28,114]:

- **For the pooled effect (**$\boldsymbol{\mu}$**):**

$$\mu\sim N\left( 0,1 \right)$$

This prior assumes that the true log odds ratio (or hazard ratio) falls within the range of

[-2, 2] with 95% probability. This range is conservative yet broad enough to cover clinically plausible associations between biomarkers and hip fracture outcomes without assuming the direction of the effect.

- **For the heterogeneity (**$\boldsymbol{\tau}$**):**

$$\tau\sim Half-Cauchy\left( 0,0.5 \right)$$

The Half-Cauchy distribution is preferred over the inverse-gamma distribution for variance parameters in hierarchical models. Setting the scale parameter to 0.5 allows for the presence of heterogeneity (avoiding the zero-heterogeneity trap) while penalizing unrealistically large variance values.

**4. Implementation:**

The analysis was implemented using the brms package [35] in R, which utilizes the Hamiltonian Monte Carlo (HMC) algorithm (Stan).To ensure robust convergence and reliable posterior estimation, we ran 4 chains with 10,000 iterations each (including 5,000 warmup iterations).To prevent divergent transitions, we increased the target average acceptance probability to 0.999. Convergence was assessed using the potential scale reduction factor $\hat{R}$, with $\left( \hat{R}<1.01 \right)$ [115] considered indicative of convergence.We reported the posterior mean and 95% credible intervals (CrIs) for all pooled estimates.

# 5. Pre-defined Quantitative Criteria for GRADE Assessment

To minimize inter-rater subjectivity and ensure a highly reproducible evaluation of evidence certainty, our GRADE assessment was conducted using a pre-defined, quantitative framework. The certainty of evidence for each biomarker was downgraded by one level if it met the following specific statistical or numerical thresholds.

**1. Risk of Bias:**

Downgraded if studies classified as having a "high risk of bias" contributed to >60% of the cumulative statistical weight in the traditional random-effects model. This threshold ensures we downgrade only when the overall effect is predominantly driven by low-quality evidence.

**2. Inconsistency:**

Downgraded if the I^2^ statistic was >60%.Based on the Cochrane Handbook guidelines, this cutoff was selected as a definitive marker for substantial and potentially unexplained heterogeneity among the included studies.

**3. Imprecision:**

To rigorously address the risk of false-positive narrow confidence intervals, we conducted the tests using three methods. Rate down by one level if both the Knapp-Hartung 95% confidence interval (CI) and the Bayesian 95% credible interval (CrI) overlap with the null effect. Rate down by two levels if the DerSimonian-Laird, Knapp-Hartung, and Bayesian all overlap with the null effect.

**4. Publication Bias:**

Instead of relying solely on the visual inspection of funnel plot asymmetry, we employed the non-parametric trim-and-fill method (L0 estimator). The evidence was downgraded only if the adjusted pooled effect size demonstrated a relative reduction of ≥20% compared to the original DerSimonian-Laird estimate. This threshold targets clinically meaningful bias rather than minor statistical fluctuations.

**5. Indirectness:**

Not downgraded across all biomarkers.The populations, interventions, comparisons, and outcomes of the included studies directly and strictly matched the targeted clinical question of this review.

# 6. Table S2

Table S2. Definitions and calculation formulas of the evaluated composite biomarkers.

| **Category** | **Abbreviation** | **Full name** | **Calculation formula** |
| --- | --- | --- | --- |
| Inflammatory-Nutritional Indices | PNI | Prognostic nutritional index | ALB (g/dL) × 10 + TLC (/mm^3^) × 0.005 |
|  | CAR | C-reactive protein to albumin ratio | CRP (mg/dL)/ALB (g/dL) |
|  | RAR | Red cell distribution width to albumin ratio | RDW (%)/ALB (g/dL) |
|  | HRR | Hemoglobin to red cell distribution width ratio | HB (g/dL)/RDW (%) |
|  | HALP | Hemoglobin, Albumin, Lymphocyte and Platelet score | HB (g/L) × ALB (g/L) × LYM (10^9^/L)/PLT (10^9^/L) |
|  | CONUT | Controlling nutritional status score | Calculated by summing the assigned scores of serum ALB (g/dL), TLC (mm^3^), and TCL (mg/dL) score. |
| Immune and Systemic Inflammatory Indices | NLR | Neutrophil to lymphocyte ratio | NEU (/L)/LYM (/L) |
|  | SII | Systemic Immune-Inflammation Index | PLT (/L) × NEU (/L)/LYM (/L) |
|  | MLR | Monocyte to lymphocyte ratio | MONO (10^9^/L)/LYM (10^9^/L) |
|  | PLR | Platelet to lymphocyte ratio | PLT (/L)/LYM (/L) |
|  | CLR | C-reactive protein to lymphocyte ratio | CRP (mg/L)/LYM (10^9^/L) |
| Basic Nutritional and Metabolic Indices | GNRI | Geriatric nutritional risk index | 1.489 × ALB (g/L) + 41.7 × body weight/ideal body weight |
|  | GAR | Glucose to albumin ratio | Glucose (mmol/L)/ALB (g/L) |

ALB, Albumin; CRP, C-reactive protein; HB, Hemoglobin; LYM, Lymphocyte; MONO, Monocyte; NEU, Neutrophil; PLT, Platelet; RDW, Red blood cell distribution width; TCL, Total cholesterol; TLC, Total lymphocyte count.

# 7. Figure S1. Risk of bias assessments for the 65 studies included in the narrative review.

**Figure S1.** Risk of bias assessments for the 65 studies included in the narrative review. This was evaluated using the Quality in Prognosis Studies (QUIPS) tool. The risk of bias levels of low, moderate, and high are color-coded green, yellow, and red, respec-tively.

# 8. Table S3

Table S3. GRADE evidence profile and certainty of evidence for the evaluated composite biomarkers.

| **Biomarker** | **Phase** | **Study limitations** | **Inconsistency** | **Indirectness** | **Imprecision** | **Publication Bias** | **Moderate/large effect size** | | **Dose effect** | **Overall quality** |
| --- | --- | --- | --- | --- | --- | --- | --- | --- | --- | --- |
| **Short-term (OR)** | | | | | | | | | | |
| NLR | No downgrade | No downgrade | Downgrade by one level | No downgrade | No downgrade | Downgrade by one level | | No upgrade | No upgrade | Low |
| NLR* | No downgrade | No downgrade | Downgrade by one level | No downgrade | No downgrade | No downgrade | | No upgrade | No upgrade | Moderate |
| PNI | No downgrade | No downgrade | No downgrade | No downgrade | No downgrade | No downgrade | | No upgrade | No upgrade | High |
| PNI* | No downgrade | No downgrade | No downgrade | No downgrade | No downgrade | No downgrade | | No upgrade | No upgrade | High |
| CAR | No downgrade | Downgrade by one level | No downgrade | No downgrade | No downgrade | No downgrade | | No upgrade | No upgrade | Moderate |
| GAR* | No downgrade | No downgrade | No downgrade | No downgrade | No downgrade | No downgrade | | No upgrade | No upgrade | High |
| **Medium-to-long-term (OR)** | | | | | | | | | | |
| NLR* | No downgrade | Downgrade by one level | Downgrade by one level | No downgrade | No downgrade | No downgrade | | No upgrade | No upgrade | Low |
| MLR* | No downgrade | Downgrade by one level | Downgrade by one level | No downgrade | No downgrade | Downgrade by one level | | No upgrade | No upgrade | Very Low |
| GNRI* | No downgrade | Downgrade by one level | Downgrade by one level | No downgrade | No downgrade | No downgrade | | No upgrade | No upgrade | Low |
| CAR* | No downgrade | Downgrade by one level | No downgrade | No downgrade | No downgrade | No downgrade | | No upgrade | No upgrade | Moderate |
| **Medium-to-long-term (HR)** | | | | | | | | | | |
| NLR | No downgrade | No downgrade | No downgrade | No downgrade | Downgrade by two levels | No downgrade | | No upgrade | No upgrade | Low |
| SII* | No downgrade | No downgrade | Downgrade by one level | No downgrade | No downgrade | No downgrade | | No upgrade | No upgrade | Moderate |

[*] indicates that the biomarker was analyzed based on a cut-off value; biomarkers without an asterisk were analyzed as continuous variables. CAR, C-reactive protein-to-albumin ratio; GAR, glucose-to-albumin ratio; GNRI, geriatric nutritional risk index; MLR, monocyte-to-lymphocyte ratio; NLR, neutrophil-to-lymphocyte ratio; PNI, prognostic nutritional index; SII, systemic immune-inflammation index.

# 9. Figure S2. Forest plots summarizing the prognostic value of individual composite biomarkers for postoperative outcomes.

(A)

(B)

(C)

(D)

(E)

(F)

(G)

(H)

(I)

**Figure S2.** Forest plots summarizing the prognostic value of individual composite biomarkers for postoperative outcomes. The panels detail the specific effect sizes as follows: (A) pooled odds ratio (OR) of continuous C-reactive protein to albumin ratio (CAR) for predicting short-term adverse outcomes; (B) pooled OR of dichotomous CAR* for predicting medium-to-long-term outcomes; (C) pooled OR of dichotomous geriatric nutritional risk index (GNRI*) for predicting medium-to-long-term outcomes; (D) pooled OR of dichotomous monocyte to lymphocyte ratio (MLR*) for predicting medium-to-long-term outcomes; (E) pooled OR of dichotomous neutrophil to lymphocyte ratio (NLR*) for predicting short-term outcomes; (F) pooled OR of continuous NLR for predicting short-term outcomes; (G) pooled OR of dichotomous NLR* for predicting medium-to-long-term outcomes; (H) pooled hazard ratio (HR) of continuous NLR for predicting medium-to-long-term outcomes; and (I) pooled HR of dichotomous systemic immune-inflammation index (SII*) for predicting medium-to-long-term outcomes. Green, yellow, and red indicators represent low, moderate, and high risks of bias, respectively. The biomarkers marked with an asterisk [*] represent categorical variables classified based on their respective cutoff values, while those without an asterisk were analyzed as continuous variables.

# 10. Figure S3: Sbgroup analyses of the associations between composite biomarkers and adverse outcomes

**Figure S3.** Subgroup analyses of the associations between composite biomarkers and adverse outcomes. The forest plot displays the odds ratios (ORs) and 95% confidence intervals (CIs) for short-term and medium-to-long-term outcomes across various subgroups. The red dashed vertical line represents the null effect (OR = 1). Solid squares represent the point estimates for each subgroup, with horizontal lines indicating the 95% CIs. NLR, neutrophil-to-lymphocyte ratio; MLR, monocyte-to-lymphocyte ratio; GNRI, Geriatric Nutritional Risk Index; OR, odds ratio; CI, confidence interval; Adm, admission; Pre, preoperative; Post, postoperative; POD, postoperative delirium. P for interaction indicates the significance of the difference in effect sizes between subgroups. I² represents the degree of heterogeneity within each subgroup. [*] indicates that the biomarker was analyzed based on a cut-off value; biomarkers without an asterisk were analyzed as continuous variables.

# 11. Table S4

Table S4. PRISMA 2020 checklist.

| **Section and Topic** | **Item #** | **Checklist item** | **Location where item is reported in page#** |
| --- | --- | --- | --- |
| **TITLE** | | |  |
| Title | 1 | Identify the report as a systematic review. | 1 |
| **ABSTRACT** | | |  |
| Abstract | 2 | See the PRISMA 2020 for Abstracts checklist. | 1 |
| **INTRODUCTION** | | |  |
| Rationale | 3 | Describe the rationale for the review in the context of existing knowledge. | 2 |
| Objectives | 4 | Provide an explicit statement of the objective(s) or question(s) the review addresses. | 2 |
| **METHODS** | | |  |
| Eligibility criteria | 5 | Specify the inclusion and exclusion criteria for the review and how studies were grouped for the syntheses. | 3 |
| Information sources | 6 | Specify all databases, registers, websites, organisations, reference lists and other sources searched or consulted to identify studies. Specify the date when each source was last searched or consulted. | 3 |
| Search strategy | 7 | Present the full search strategies for all databases, registers and websites, including any filters and limits used. | Supplement S1 |
| Selection process | 8 | Specify the methods used to decide whether a study met the inclusion criteria of the review, including how many reviewers screened each record and each report retrieved, whether they worked independently, and if applicable, details of automation tools used in the process. | 4 |
| Data collection process | 9 | Specify the methods used to collect data from reports, including how many reviewers collected data from each report, whether they worked independently, any processes for obtaining or confirming data from study investigators, and if applicable, details of automation tools used in the process. | 4 |
| Data items | 10a | List and define all outcomes for which data were sought. Specify whether all results that were compatible with each outcome domain in each study were sought (e.g. for all measures, time points, analyses), and if not, the methods used to decide which results to collect. | 4 |
|  | 10b | List and define all other variables for which data were sought (e.g. participant and intervention characteristics, funding sources). Describe any assumptions made about any missing or unclear information. | Supplement S6 |
| Study risk of bias assessment | 11 | Specify the methods used to assess risk of bias in the included studies, including details of the tool(s) used, how many reviewers assessed each study and whether they worked independently, and if applicable, details of automation tools used in the process. | 4 |
| Effect measures | 12 | Specify for each outcome the effect measure(s) (e.g. risk ratio, mean difference) used in the synthesis or presentation of results. | 4 |
| Synthesis methods | 13a | Describe the processes used to decide which studies were eligible for each synthesis (e.g. tabulating the study intervention characteristics and comparing against the planned groups for each synthesis (item #5)). | 3 |
|  | 13b | Describe any methods required to prepare the data for presentation or synthesis, such as handling of missing summary statistics, or data conversions. | 4 |
|  | 13c | Describe any methods used to tabulate or visually display results of individual studies and syntheses. | 4 |
|  | 13d | Describe any methods used to synthesize results and provide a rationale for the choice(s). If meta-analysis was performed, describe the model(s), method(s) to identify the presence and extent of statistical heterogeneity, and software package(s) used. | 5 |
|  | 13e | Describe any methods used to explore possible causes of heterogeneity among study results (e.g. subgroup analysis, meta-regression). | 5 |
|  | 13f | Describe any sensitivity analyses conducted to assess robustness of the synthesized results. | Supplement S4, page 4-5 |
| Reporting bias assessment | 14 | Describe any methods used to assess risk of bias due to missing results in a synthesis (arising from reporting biases). | Supplement S3, page 5 |
| Certainty assessment | 15 | Describe any methods used to assess certainty (or confidence) in the body of evidence for an outcome. | Supplement S5, page 5 |
| **RESULTS** | | |  |
| Study selection | 16a | Describe the results of the search and selection process, from the number of records identified in the search to the number of studies included in the review, ideally using a flow diagram. | Please see figure1. |
|  | 16b | Cite studies that might appear to meet the inclusion criteria, but which were excluded, and explain why they were excluded. | Please see figure1. |
| Study characteristics | 17 | Cite each included study and present its characteristics. | Supplement S6, page 5-6 |
| Risk of bias in studies | 18 | Present assessments of risk of bias for each included study. | Figure 3, supplement S9 |
| Results of individual studies | 19 | For all outcomes, present, for each study: (a) summary statistics for each group (where appropriate) and (b) an effect estimate and its precision (e.g. confidence/credible interval), ideally using structured tables or plots. | Supplement S6 |
| Results of syntheses | 20a | For each synthesis, briefly summarise the characteristics and risk of bias among contributing studies. | Supplement S8 |
|  | 20b | Present results of all statistical syntheses conducted. If meta-analysis was done, present for each the summary estimate and its precision (e.g. confidence/credible interval) and measures of statistical heterogeneity. If comparing groups, describe the direction of the effect. | Figure 4, figure 5, supplement S11, page 8-13 |
|  | 20c | Present results of all investigations of possible causes of heterogeneity among study results. | Supplement S9 |
|  | 20d | Present results of all sensitivity analyses conducted to assess the robustness of the synthesized results. | Supplement S12, supplement S13, page13-14 |
| Reporting biases | 21 | Present assessments of risk of bias due to missing results (arising from reporting biases) for each synthesis assessed. | Figure 6, supplement S14, page13-14 |
| Certainty of evidence | 22 | Present assessments of certainty (or confidence) in the body of evidence for each outcome assessed. | Supplement S10,  figure 4, page 8-14 |
| **DISCUSSION** | | |  |
| Discussion | 23a | Provide a general interpretation of the results in the context of other evidence. | 15-16 |
|  | 23b | Discuss any limitations of the evidence included in the review. | 17 |
|  | 23c | Discuss any limitations of the review processes used. | 17 |
|  | 23d | Discuss implications of the results for practice, policy, and future research. | 17 |
| **OTHER INFORMATION** | | |  |
| Registration and protocol | 24a | Provide registration information for the review, including register name and registration number, or state that the review was not registered. | 3 |
|  | 24b | Indicate where the review protocol can be accessed, or state that a protocol was not prepared. | 3 |
|  | 24c | Describe and explain any amendments to information provided at registration or in the protocol. | Not modified |
| Support | 25 | Describe sources of financial or non-financial support for the review, and the role of the funders or sponsors in the review. | 18 |
| Competing interests | 26 | Declare any competing interests of review authors. | 18 |
| Availability of data, code and other materials | 27 | Report which of the following are publicly available and where they can be found: template data collection forms; data extracted from included studies; data used for all analyses; analytic code; any other materials used in the review. | 18 |
